# Dominant Role of the p110β Isoform of PI3K over p110α in Energy Homeostasis Regulation by POMC and AgRP Neurons

**DOI:** 10.1016/j.cmet.2009.09.008

**Published:** 2009-11-04

**Authors:** Hind Al-Qassab, Mark A. Smith, Elaine E. Irvine, Julie Guillermet-Guibert, Marc Claret, Agharul I. Choudhury, Colin Selman, Kaisa Piipari, Melanie Clements, Steven Lingard, Keval Chandarana, Jimmy D. Bell, Gregory S. Barsh, Andrew J.H. Smith, Rachel L. Batterham, Michael L.J. Ashford, Bart Vanhaesebroeck, Dominic J. Withers

**Affiliations:** 1 Centre for Diabetes and Endocrinology, Rayne Institute, University College London, London WC1E 6JJ, UK; 2 Centre for Cell Signalling, Institute of Cancer, Queen Mary University of London, Charterhouse Square, London EC1M 6BQ, UK; 3 Molecular Imaging Group, Medical Research Council Clinical Sciences Centre, Imperial College, London W12 0NN, UK; 4 Department of Genetics, Stanford University, Stanford, CA 94305, USA; 5 Gene Targeting Laboratory, The Institute of Stem Cell Research, University of Edinburgh, Edinburgh EH9 3JQ, UK; 6 Biomedical Research Institute, Ninewells Hospital and Medical School, University of Dundee, Dundee DD1 9SY, UK

**Keywords:** HUMDISEASE

## Abstract

PI3K signaling is thought to mediate leptin and insulin action in hypothalamic pro-opiomelanocortin (POMC) and agouti-related protein (AgRP) neurons, key regulators of energy homeostasis, through largely unknown mechanisms. We inactivated either p110α or p110β PI3K catalytic subunits in these neurons and demonstrate a dominant role for the latter in energy homeostasis regulation. In POMC neurons, p110β inactivation prevented insulin- and leptin-stimulated electrophysiological responses. POMCp110β null mice exhibited central leptin resistance, increased adiposity, and diet-induced obesity. In contrast, the response to leptin was not blocked in p110α-deficient POMC neurons. Accordingly, POMCp110α null mice displayed minimal energy homeostasis abnormalities. Similarly, in AgRP neurons, p110β had a more important role than p110α. AgRPp110α null mice displayed normal energy homeostasis regulation, whereas AgRPp110β null mice were lean, with increased leptin sensitivity and resistance to diet-induced obesity. These results demonstrate distinct metabolic roles for the p110α and p110β isoforms of PI3K in hypothalamic energy regulation.

## Introduction

Increased understanding of the molecular and cellular mechanisms that regulate whole-body energy homeostasis is needed to gain insights into the pathophysiology of obesity and for the development of effective treatments (Barsh et al., 2000; Barsh and Schwartz, 2002; Morton et al., 2006). Hypothalamic arcuate nucleus (ARC) pro-opiomelanocortin (POMC)-expressing neurons, and agouti-related protein (AgRP)- and neuropeptide Y (NPY)-expressing neurons sense peripheral and central signals that reflect nutritional status responding to nutrients, anorexigenic peripheral hormones such as leptin and insulin, and centrally derived neuropeptides and neurotransmitters (Barsh et al., 2000; Barsh and Schwartz, 2002; Bewick et al., 2005; Gropp et al., 2005; Luquet et al., 2005; Xu et al., 2005a; Choudhury et al., 2005; Claret et al., 2007; Morton et al., 2006; Smith et al., 2007). Integration of these signals by POMC and AgRP/NPY neurons regulates both their neuronal activity and the expression and release of their cognate neuropeptides and other neurotransmitters, which combine to control both short- and long-term energy balance (Barsh et al., 2000; Barsh and Schwartz, 2002).

In these neurons, the precise intracellular signaling machinery upon which both leptin and insulin act is incompletely defined. Recent attention has focused upon class IA phosphoinositide 3-kinases (PI3Ks), which are acutely regulated by extracellular stimuli and have pleiotropic roles in cellular and organismal physiology (Vanhaesebroeck et al., 2005). Class IA PI3K isoforms consist of a p110 catalytic subunit (p110α, p110β, or p110δ) constitutively bound to one of five distinct p85 regulatory subunits (Vanhaesebroeck et al., 2005). p110α and p110β are widely expressed, while p110δ is predominantly expressed in leucocytes (Vanhaesebroeck et al., 2005). Class IA PI3Ks catalyze the synthesis of the lipid second messenger phosphatidylinositol (3,4,5)-triphosphate (PIP3), which engages downstream effectors such as the protein kinase B (PKB) pathway (Shepherd et al., 1998; Vanhaesebroeck et al., 2005).

Evidence has implicated class IA PI3Ks in hypothalamic function, suggesting that they are a point of signaling integration for leptin and insulin action. Both hormones stimulate PI3K activity in mediobasal hypothalamic lysates and PIP3 production in POMC neurons (Niswender et al., 2001, 2003). Pharmacological inhibition of PI3K activity using broad-spectrum PI3K inhibitors blocks the electrophysiological effects of leptin and insulin on POMC neurons and inhibits the acute effects of leptin upon feeding and glucose homeostasis (Hill et al., 2008; Morton et al., 2005). However, there are significant unanswered questions regarding the precise role of class IA PI3K isoforms in the hypothalamic regulation of energy homeostasis. First, the effects of specific long-term manipulation of hypothalamic expression of the two major catalytic subunits, p110α and p110β, on body weight regulation are not known. Recent pharmacological evidence using isoform-specific PI3K inhibitors and genetic studies using conditional p110α and p110β null mice and cells have started to reveal specific roles for p110α and p110β in peripheral tissues (Chaussade et al., 2007; Ciraolo et al., 2008; Graupera et al., 2008; Jia et al., 2008; Knight et al., 2006). Therefore, these molecules may have different contributions to the regulation of neuronal function. The role of PI3K signaling in AgRP neurons in the regulation of energy homeostasis has also not been determined. In the context of ongoing PI3K drug development, a key question is which of the many PI3K isoforms should be targeted to achieve specific therapeutic benefit (Marone et al., 2008; Wymann et al., 2003). We therefore inactivated p110α and p110β in POMC or AgRP neurons to determine the role of these kinases in energy homeostasis.

## Results

### Generation of Mice Lacking p110α or p110β in POMC and AgRP Neurons

**Figure 1:**
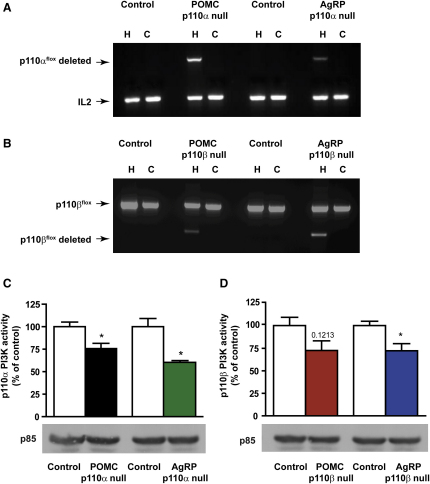
Genetic Inactivation of p110α and p110β in Hypothalami of POMCp110 Null and AgRPp110 Null Mice Recombination of p110α (A) or p110β (B) alleles in the hypothalamus (H), but not the cerebral cortex (C), of POMCp110null and AgRPp110 null mice. Unaltered expression of p85 and reduction of p110α activity (C) or p110β activity (D) in hypothalamic lysates from POMCp110 null and AgRPp110 null mice, n = 3. All values are mean ± SEM, ^∗^p < 0.05.

Mice with floxed alleles of either p110α (*Pik3ca*) or p110β (*Pik3cb*) (Graupera et al., 2008; Guillermet-Guibert et al., 2008) were crossed with mice that express Cre recombinase in POMC or AgRP neurons (Choudhury et al., 2005; Claret et al., 2007; Xu et al., 2005a, 2005b) to generate POMCp110 null and AgRPp110 null mice for each isoform and relevant control strains. The floxed alleles of p110α and p110β were designed to preserve the signaling stoichiometry of the p85/p110 PI3K signaling complexes (Graupera et al., 2008; Guillermet-Guibert et al., 2008). In the brain, genetic inactivation of p110α or p110β was restricted to the hypothalamus, as determined by PCR analysis of the recombination event (Figures 1A and 1B). We did not detect Cre recombinase expression in the dentate gyrus or nucleus tractus solitarus (data not shown). Hypothalamic p110α lipid kinase activity in POMC- and AgRPp110α null mice (Figure 1C) and p110β activity in POMC- and AgRPp110β null mice (Figure 1D) was reduced, but expression of p85 was unaltered (Figures 1C and 1D). Expression of p110α, p110β, and p85 in muscle, liver, and fat was also equivalent in mutant and control mice (data not shown).

### Inactivation of p110α or p110β in POMC and AgRP Neurons Does Not Lead to Observable Alterations in Cell Body Organization and Number

PI3K signaling plays key roles in cellular function, but mutant mice did not show alterations in the location, population size, or somatic dimensions of POMC (see Figures S1A–S1H available online) or AgRP neurons (Figures S2A–S2H) compared to control mice. No differences were observed in basic neuronal biophysical properties in the mutant mice, although the resting membrane potential of POMCp110α null neurons was slightly hyperpolarized compared to control POMC neurons, with a concomitant reduction in firing rate (Table S1). However, this change in firing rate did not affect basal peptide release, as hypothalamic explant studies showed that release of alpha melanocyte-stimulating hormone (α-MSH) and AgRP in POMC- and AgRP-targeted mutants, respectively, was equivalent to control mice (Figures S3A–S3D). The POMC promoter also drives Cre recombinase expression in anterior pituitary corticotrophs, but corticosterone levels in all four mutant lines were equivalent to control mice (Figures S4A–S4D).

### POMCp110β Null Mice Display Increased Food Intake, Adiposity, and Sensitivity to a High-Fat Diet

**Figure 2:**
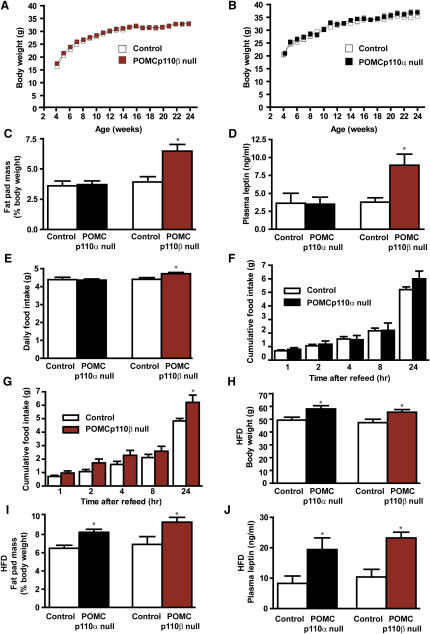
Energy Homeostasis Phenotypes in POMCp110α Null and POMCp110β Null Mice Body weight curves of male POMCp110β null (A) and POMCp110α null (B) mice on chow diet, n = 30 per genotype. (C) Fat pad mass in 40-week-old POMCp110α null and POMCp110β null mice, n = 10. (D) Fasting plasma leptin levels in control, POMCp110α null, and POMCp110β null mice, n = 8. (E) Twenty-four hour food intake under freely feeding conditions in 12-week-old male control, POMCp110α null and POMCp110β null mice, n = 10–14. Food intake after overnight fast in 16-week-old male POMCp110α null (F) and POMCp110β null (G) mice, n = 10–14. Body weight (H), percentage fat mass (I), and fasting plasma leptin levels (J) of POMCp110α null and POMCp110β null mice following 18 week exposure to HFD, n = 10–15. All values are mean ± SEM, ^∗^p < 0.05.

POMCp110β null mice on standard chow displayed normal total body mass but an increased fat mass and fasting hyperleptinemia (Figures 2A, 2C, and 2D). Magnetic resonance imaging (MRI) at 24 weeks of age confirmed increased total body adiposity (fat mass per body weight, POMCp110β null 16.2% ± 1.2% versus control 9.5% ± 1.3%, n = 5, p < 0.01). POMCp110α null mice, in contrast, displayed normal body weight, fat mass, and leptin levels (Figures 2B–2D). POMCp110β null mice, but not POMCp110α null mice, displayed increased food intake, both daily and following an overnight fast (Figures 2E–2G), and increased linear growth (Figures S5A and S5B). Resting metabolic rate (RMR) and sensitivity to the peripherally administered MC3/4R agonist melanotan II (MT-II) were normal in both POMCp110α null and POMCp110β null mice (Figures S5C–S5F). On a high-fat diet (HFD), both POMCp110α null and POMCp110β null mice displayed increased body weight, adiposity, and hyperleptinemia, compared to controls (Figures 2H–2J).

### AgRPp110β Null Mice Are Hypophagic, Lean, and Resistant to Diet-Induced Obesity

**Figure 3:**
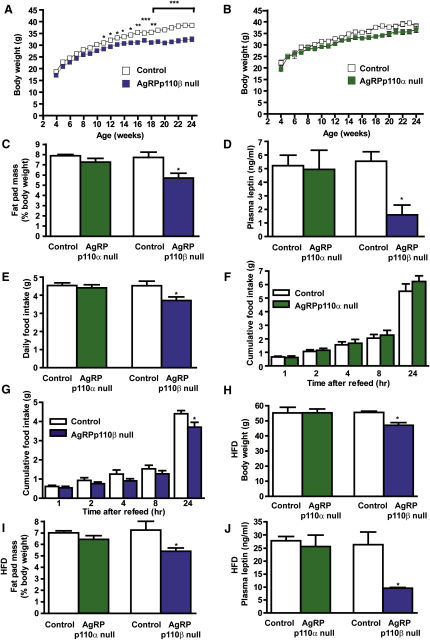
Energy Homeostasis Phenotypes in AgRPp110α Null and AgRPp110β Null Mice Body weight curves of male AgRPp110β null (A) and AgRPp110α null (B) mice on chow diet, n = 30 per genotype. (C) Percentage fat pad mass in 40-week-old AgRPp110α null and AgRPp110β null mice, n = 10. (D) Fasting plasma leptin levels in AgRPp110α null and AgRPp110β null mice, n = 8. (E) Twenty-four hour food intake under freely feeding conditions in 12-week-old male AgRPp110α null and AgRPp110β null mice, n = 10–14. Food intake after overnight fast in 16-week-old male AgRPp110α null (F) and AgRPp110β null (G) mice, n = 10–12. Body weight (H), percentage fat mass (I), and fasting plasma leptin levels (J) of AgRPp110α null and AgRPp110β null mice following 18 week exposure to HFD, n = 10–15. All values are mean ± SEM, ^∗^p < 0.05, ^∗∗^p < 0.01, ^∗∗∗^p < 0.001.

Body weight (Figure 3A), fat mass (Figure 3C), and leptin levels (Figure 3D) were significantly lower in AgRPp110β null mice. Food intake ad libitum and following an overnight fast was reduced in AgRPp110β null mice (Figures 3E and 3G). In contrast, AgRPp110α null mice displayed no significant alterations within these parameters (Figures 3B–3F). RMR and sensitivity to MT-II were normal in both AgRPp110α null and AgRPp110β null mice (Figures S6A–S6D). On HFD, AgRPp110β null, but not AgRPp110α null, mice displayed a significant reduction in body weight, fat mass, and leptin levels, compared to controls (Figures 3H–3J).

### Leptin-Mediated Suppression of Food Intake Is Impaired in POMCp110β Null Mice but Enhanced in AgRPp110β Null Mice

**Figure 4:**
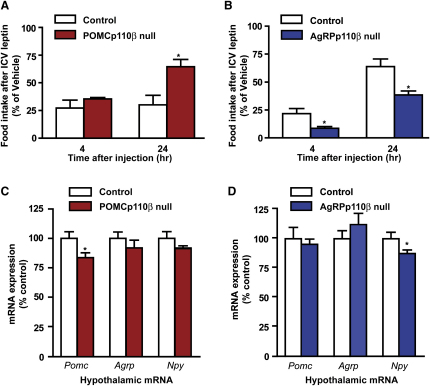
Response to Centrally Administered Leptin and Hypothalamic Neuropeptide Expression of POMCp110β Null and AgRPp110β Null Mice Suppression of food intake in POMCp110β null (A) and AgRPp110β null (B) mice following i.c.v. injection of leptin (0.5 μg), n = 8. Pomc, Agrp, and Npy mRNA expression in hypothalami of POMCp110β null (C) and AgRPp110β null (D) mice. All values are mean ± SEM, ^∗^p < 0.05, ^∗∗∗^p < 0.001.

In POMCp110β null mice, suppression of food intake by leptin administered into the third cerebral ventricle (i.c.v.) was equivalent to control mice at 4 hr but blunted at 24 hr postinjection (Figure 4A). Conversely, AgRPp110β null mice had increased sensitivity to i.c.v. leptin at both 4 and 24 hr postinjection, compared to controls (Figure 4B). Inactivating p110α in POMC or AgRP neurons did not affect the response to leptin (Figures S7A and S7B).

### Hypothalamic Neuropeptide mRNA Expression and Glucose Homeostasis in p110 Mutant Mice

A small but significant reduction in *Pomc* mRNA was detectable in fasted POMCp110β null mice, while *Agrp* and *Npy* mRNA were unaltered (Figure 4C). *Npy* mRNA was reduced in AgRPp110βnull mice, while *Pomc* and *Agrp* mRNA were unchanged (Figure 4D). No differences in the expression of *Pomc*, *Agrp*, and *Npy* mRNA were detected in POMCp110α null and AgRPp110α null mice (Figures S7C and S7D). Leptin also recruits the janus kinase/signal transducer and activator of transcription (JAK/STAT) pathway to modulate the expression of arcuate neuropeptides. However, we found no alteration of leptin-stimulated STAT3 phosphorylation in POMC or AgRP neurons lacking p110β (percent leptin-stimulated POMC or AgRP neuron pSTAT3: control, 46% ± 6% versus POMC p110β null, 42% ± 5%, p = N.S.; control 54% ± 5% versus AgRP p110β null 67% ± 9% p = N.S., n = 3 animals per genotype and Figure S8). POMC and AgRP neurons have been implicated in the central regulation of glucose homeostasis (Konner et al., 2007; Parton et al., 2007), but no alterations were found in fasting glucose levels, glucose tolerance, and fasting insulin levels in all four mutant lines (Figures S9A–S9H).

### p110β Is Required for Leptin-Induced Depolarization of POMC Neurons

**Figure 5:**
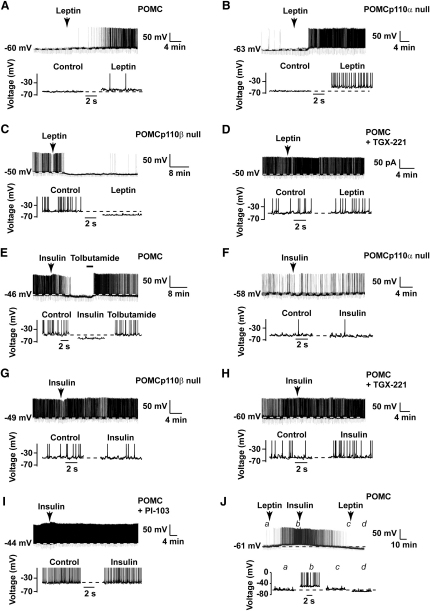
PI3K Activity Underlies Leptin and Insulin Modulation of POMC Neuronal Excitability Whole-cell recordings were made from control (A, D, E, H, I, and J), p110α null (B and F), and p110β null (C and G) POMC neurons. Continuous current-clamp traces are shown in upper traces and expanded sections in lower traces, respectively. A minority population of control (A) and p110α null (B) POMC neurons were depolarized by leptin (50 nM for 2 min, as indicated by the arrows), which was associated with an increase in spike firing frequency (upward deflections). Leptin hyperpolarized a minority population of p110β null (C) but had no effect on the majority of p110β-inhibited (1 μM TGX-221) (D) POMC neurons. (E) Insulin (50 nM for 2 min, where indicated) hyperpolarized the majority of POMC neurons, which was reversed by the subsequent application of 200 μM tolbutamide. Note that there was a small reduction in input resistance following insulin application, as denoted by the reduced amplitude of the periodic downward deflections shown in the continuous trace. Genetic inactivation of p110α (F) or p110β (G) prevented insulin modulation of POMC neuron excitability. Pharmacological inhibition of p110β (1 μM TGX-221) (H) or a general PI3K inhibitor (100 nM PI-103) (I) prevented insulin modulation of POMC neuronal excitability. (J) Representative continuous current-clamp trace before and after sequential leptin and insulin (50 nM for 2 min) application as indicated by the arrows. Expanded sections are shown underneath at time points indicated by the corresponding letters in italics. Note that leptin-induced depolarization was reversed by subsequent insulin application, although a subsequent leptin administration had no effect on membrane potential.

We next used electrophysiological analysis to investigate neuronal responses to leptin and insulin in POMCp110β null and POMCp110α null mice. Consistent with previous observations (Choudhury et al., 2005; Claret et al., 2007; Cowley et al., 2001; Plum et al., 2006), a subpopulation of control POMC neurons (6 of 21) responded to locally applied leptin (50 nM) by long-lasting (>1 hr) membrane depolarization (Figure 5A), an action significant for the recorded population (n = 21, p < 0.05; Table 1). Although leptin depolarization of POMC neurons could be observed at resting membrane potentials (Vm) of approximately −50 mV, the magnitude of response was greater at more hyperpolarized Vm (r^2^ = 0.63, n = 21, p < 0.0001; Figure S10A). As previously reported, the majority of control POMC neurons were unresponsive to leptin (Table 1 and Figure S10B).

As POMCp110β null mice displayed reduced sensitivity to leptin, we examined the effect of genetic inactivation of specific PI3K catalytic subunit isoforms on leptin-mediated POMC excitability. In mice aged 8–16 weeks, leptin depolarized (n = 13, p < 0.05) the POMCp110α null neuronal population and increased their spike firing frequency (Figure 5B and Table 1), in agreement with the unchanged leptin sensitivity observed in vivo. In contrast, leptin did not depolarize POMCp110β null neurons, and indeed many POMCp110β null neurons (7 of 17) exhibited long-lasting hyperpolarization following leptin application (Figure 5C, Table 1). Subsequently, in a separate series of experiments on age- (7- and 18-week-old) and sex-matched POMCp110β null mutant mice, we first showed that food intake was elevated at both 7 and 18 weeks of age. Irrespective of age or metabolic phenotype, subsequent electrophysiological recordings from POMCp110β null neurons demonstrated that there were no alterations to POMC neuron resting membrane potential, spike firing frequency, or input resistance in comparison to littermate control POMC neurons (Table S2). Furthermore, these POMCp110β null neurons exhibited the same altered response to leptin (i.e., conversion of depolarization to hyperpolarization) that was significantly different from the leptin-mediated excitation of control POMC neurons.

To exclude the possibility that compensatory changes associated with chronic ablation of p110β expression were responsible for this altered leptin response, in separate experiments on control POMC neurons, the selective p110β inhibitor TGX-221 (1 μM) (Jackson et al., 2005) was added to the internal pipette solution. Following a minimum of 10 min of intracellular dialysis, leptin (50 nM) application did not excite TGX-221-treated POMC neurons (n = 11, N.S.; Figure 5D and Table 1). On one occasion, a large leptin-mediated hyperpolarization was observed, which was reversibly occluded by bath-applied tolbutamide, indicating the likely involvement of ATP-sensitive K^+^ (K_ATP_) channels in this hyperpolarizing response (Figure S10C). Overall, these electrophysiological outcomes reflect the decreased leptin sensitivity found in POMCp110β null mice in vivo.

### Inactivation of p110α or p110β in POMC Neurons Prevents Insulin-Induced Hyperpolarization

POMC neurons are also targets for insulin action, and consistent with previous reports (Choudhury et al., 2005; Claret et al., 2007; Hill et al., 2008; Konner et al., 2007; Plum et al., 2007), a subpopulation of control POMC neurons (12 of 19) responded to insulin by long-lasting (>1 hr) hyperpolarization (Table 1 and Figure 5E). Subsequent bath application of tolbutamide (200 μM) reversed this response (Figure 5E). The remaining neurons were unresponsive to insulin (50 nM, Figure S10D). Neither POMCp110α null (n = 8, N.S.; Figure 5F) nor POMCp110β null (n = 9, N.S.; Figure 5G) neurons responded to insulin (Table 1), suggesting that both p110 isoforms contribute to the action of insulin in POMC neurons. Acute pharmacological inhibition of p110β by TGX-221 (1 μM) also prevented insulin hyperpolarization of POMC neurons (n = 8, N.S.; Figure 5H and Table 1). In addition, the pan-PI3K/mTOR inhibitor, PI-103 (100 nM), prevented insulin-evoked POMC neuron hyperpolarization (n = 7; Figure 5I and Table 1).

Thus, leptin and insulin induce opposing electrical responses in subsets of POMC neurons, and both outcomes require the p110β subunit of PI3K. This result could be due to differential expression of the hormone receptors and/or of the p110α and p110β subunits in the POMC neuron population. We attempted to resolve this issue in two ways, by electrophysiological analysis of the actions of sequentially applied leptin and insulin to single POMC neurons and by immunohistochemical detection of POMC neurons in mice expressing lacZ from either the p110α or the p110β loci. Sequential leptin- and insulin-induced depolarization and hyperpolarization, respectively, were observed in three of eight recordings, indicating functional colocalization of receptors in a subpopulation of POMC neurons (Figure 5J and Figure S11A). However, some POMC neurons only responded to leptin (two of eight), with the remainder (three of eight) not responding to either hormone. Note that we did not observe POMC neurons that only responded to insulin. Furthermore, 47% (±4.3, n = 3) of POMC neurons expressed p110β and 55% (±5.7, n = 3) p110α (Figures S11B and S11C). Accordingly, some POMC neurons must express both hormone receptors and p110 subunits, whereas the expression of p110β alone may explain why some POMC neurons respond only to leptin.

### p110α and p110β Are Required for Insulin-Induced Depolarization of AgRP Neurons

**Figure 6:**
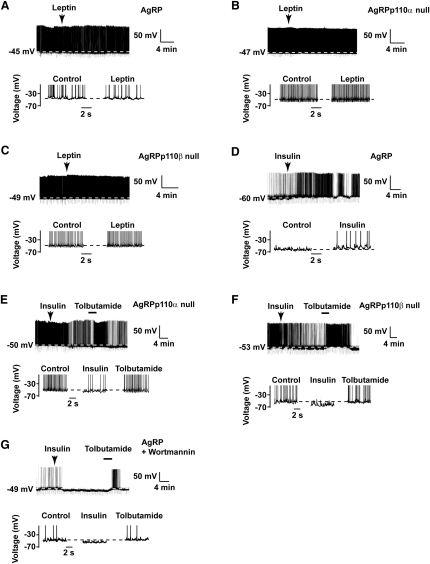
Insulin-Induced Depolarization of AgRP Neurons Is Dependent on p110α and p110β Expression Leptin (50 nM) does not modulate the excitability of control (A) and p110α (B) or p110β (C) null AgRP neurons. (D) A minority population of AgRP neurons were depolarized by insulin (50 nM), and this was associated with an increase in spike firing frequency. A proportion of p110α (E) and p110β (F) null or PI3K-inhibited (100 nM wortmannin; G) AgRP neurons were hyperpolarized by insulin, and this effect was reversibly occluded by 200 μM tolbutamide.

Consistent with our previous data (Claret et al., 2007), leptin (50 nM) did not affect Vm or spike frequency in control AgRP (n = 7, N.S., Table 1, Figure 6A), or AgRPp110α null (n = 7, N.S.; Table 1, Figure 6B), or AgRPp110β null (n = 7, N.S.; Table 1, Figure 6C) neurons. In contrast, insulin (50 nM) caused a long-lasting (>1 hr) depolarization of a subpopulation (4 of 13) of control AgRP neurons (Figure 6D), an action significant for the recorded population (n = 13, p < 0.05; Table 1). As observed for leptin on POMC neurons, the depolarization induced by insulin was greater at more hyperpolarized Vm (r^2^ = 0.65, p < 0.0001, n = 13, Figure S12A). Insulin did not change the excitability of the remaining AgRP neurons (recording periods up to 1 hr; Figure S12B). We next tested whether inactivation of p110α or p110β prevented or modified insulin action on AgRP neurons. Surprisingly, insulin hyperpolarized both AgRPp110α null (n = 7, p < 0.05; Figure 6E) and AgRPp110β null (n = 11, p < 0.05; Figure 6F) neurons, and these responses were reversed by tolbutamide. In addition, the pan-PI3K inhibitor wortmannin (100 nM present in the internal recording solution), although having no effect per se on Vm, also resulted in insulin hyperpolarizing control AgRP neurons (n = 10, p < 0.05; Table 1), a response occluded, reversibly, by tolbutamide (Figure 6G).

## Discussion

Hypothalamic PI3K signaling mechanisms have received significant attention and may act as a point of convergence for leptin and insulin action in POMC and AgRP neurons. However, studies to date have indirectly used genetics to manipulate PI3K signaling or have used inhibitors such as wortmannin and LY294002, which inhibit all PI3K isoforms and several other kinases such as mTOR, which has also been implicated in hypothalamic function. Both strategies also do not enable discrimination between the distinct PI3K isoforms. Using newly created floxed alleles of p110α and p110β, which preserve the stoichiometry of the class IA PI3K signaling network (Graupera et al., 2008; Guillermet-Guibert et al., 2008), our study reveals a dominant role for p110β in POMC and AgRP neurons.

Our studies (summarized in Table S3) show that POMCp110β null mice display hyperphagia, increased adiposity, and hyperleptinemia on normal chow diet and increased sensitivity to high-fat feeding. Furthermore, POMC neurons, in which p110β was genetically or pharmacologically inhibited, were electrically unresponsive to insulin and leptin. Indeed, the inability of leptin to excite POMCp110β null neurons (and instead hyperpolarize them) was correlated with elevated food intake in POMCp110β null mice, which was also not fully suppressed by centrally administered leptin. POMCp110β null mice had a reduction in POMC mRNA levels consistent with reports suggesting that the modulation of POMC transcription by leptin is dependent on PI3K-controlled regulation of forkhead box O1 (FoxO1) activity (Kim et al., 2006; Kitamura et al., 2006). Thus, inactivation of p110β in POMC neurons may result in an overall reduction in the expression and release of α-MSH, which may make these mice more likely to store excess energy as fat on a standard chow diet and more susceptible to weight gain when exposed to a HFD. In contrast, POMCp110α null mice did not display a significant body weight phenotype under standard chow conditions but did develop increased adiposity and hyperleptinemia when exposed to a HFD, indicating an as yet undefined role for POMCp110α in response to excess caloric intake. Genetic inactivation of p110α prevented insulin-induced hyperpolarization, although it did not prevent leptin-mediated depolarization of POMC neurons. The precise roles of insulin action on this neuronal type have not been fully elucidated, but as deletion of the insulin receptor on POMC neurons is not reported to cause a metabolic phenotype (Konner et al., 2007), the lack of phenotype in POMCp110α mice is consistent with this observation. Blockade of insulin-induced hyperpolarization in POMC neurons may therefore not translate to abnormalities in metabolism.

Previous mouse genetic evidence has also linked PI3K signaling in POMC neurons with the regulation of energy homeostasis. However, due to the nature of the various approaches, there has been some discordance in the results. For example, PIP3 production was elevated in POMC neurons in which the phosphatase and tensin homolog (Pten), a PIP3 phosphatase, was specifically deleted, and these mice were hyperphagic and displayed diet-sensitive obesity (Plum et al., 2006). Mice harboring combined POMC-selective deletion of p85α with a global deletion of p85β subunit were unresponsive to insulin and leptin in electrophysiological studies (Hill et al., 2008). These mice had abnormalities in both short-term feeding and acute responses to leptin but had no long-term disorder in energy homeostasis. However, POMC-specific deletion of *Pten* or p85 is likely to have effects beyond PI3K signaling. Deletion of *Pten* has a profound anatomical impact on POMC neurons, and the role of both Pten's lipid phosphatase (i.e., PI3K-dependent) and protein phosphatase activities in regulating leptin action suggest that this model may have additional abnormalities (Ning et al., 2006; Plum et al., 2006). Deletion of the p85 regulatory subunits often leads to increased PI3K signaling (Vanhaesebroeck et al., 2005). For example, the p85β global null mouse has improved insulin and potentially leptin action and is smaller than control littermates (Ueki et al., 2002). Furthermore, tyrosine phosphorylation of IRS2 is upregulated in these mice, which may impact upon long-term energy balance. p85α and p85β subunits also have signaling roles independent of their association with p110 catalytic subunits, and therefore full deletion of p85 subunits may lead to effects independent of PI3K catalytic activity (Okkenhaug and Vanhaesebroeck, 2001).

Like POMC neurons, AgRP neurons are targets for leptin and insulin action, but the role of PI3K in these neurons is largely unknown. Mice in which p110β, but not p110α, was inactivated in AgRP neurons display an age-dependent lean phenotype with reduced adiposity, hypoleptinemia, and resistance to diet-induced obesity (Table S3). This phenotype may be surprising, given that disruption of leptin receptor expression specifically in AgRP neurons results in mild obesity, suggesting that loss of leptin action via PI3K signaling might result in a similar phenotype. However, leptin withdrawal, rather than administration, has been described to activate PI3K and accumulate PIP3 in AgRP neurons (Xu et al., 2005b). Thus reducing PI3K activity in AgRP neurons may result in these cells behaving as if they are exposed to increased leptin levels, physiologically resulting in a lean phenotype (Xu et al., 2005b). However, consistent with our previous study (Claret et al., 2007), leptin did not change the excitability of control and p110α or p110β null AgRP neurons. Although others have observed leptin-mediated hyperpolarization of a subpopulation of rat AgRP-expressing neurons (van den Top et al., 2004), methodological and species differences may explain these discrepancies. Nevertheless, insulin excites AgRP neurons under our recording conditions (Claret et al., 2007), but in p110β- or p110α-deleted as well as pharmacologically PI3K-inhibited AgRP neurons, insulin inhibits the excitability of these neurons. The net effect of this alteration would be to reduce insulin-induced depolarization and consequently decrease the release of AgRP and NPY with a resultant attenuation in orexigenic output. Mice lacking either *Agrp* or *Npy* have mild body weight phenotypes or reduced feeding after a fast (Patel et al., 2006; Wortley et al., 2005). *Npy* mRNA was reduced in the hypothalamus of AgRPp110β null mice, therefore potentially contributing to the lean phenotype observed in these mice. STAT3 signaling is reported to play a major role in mediating leptin-induced alteration in neuropeptide expression including *Agrp* and *Npy*. However, leptin-stimulated STAT3 phosphorylation was equivalent in both AgRP and AgRPp110β null neurons and is unlikely to underlie the lean phenotype in these mice.

An important issue arising from the electrophysiological studies pertains to the mechanisms by which leptin and insulin, through the increased production of a signaling molecule in common (i.e., PIP3), produce opposing electrical responses in POMC neurons. Approximately half of POMC neurons express p110α or p110β. However, due to the lack of suitable reagents, we have not been able to establish the coincidence of expression of these subunits in POMC neurons. The finding that leptin, but not insulin, alone can modify electrical activity of some POMC neurons could be explained either by selective expression of the p110β subunit alone or the leptin receptor exhibiting selective coupling to this subunit. In contrast, it is less likely that p110α is expressed in a subpopulation of POMC neurons independently from p110β, as insulin responses were only observed in neurons that also responded to leptin and insulin-mediated POMC neuron hyperpolarization required the presence of both p110 subunits. The inability of both leptin and insulin to modulate the electrical activity of some POMC neurons may indicate that they lack functional receptors for either hormone (Cheung et al., 1997; Elias et al., 1999; Huo et al., 2009). Alternatively, these unresponsive POMC neurons may lack the p110β subunit, although we have no way of determining whether the p110α subunit is present or not in these neurons. The finding that insulin-mediated hyperpolarization of POMC, and depolarization of AgRP, neurons was ablated by removal of either p110α or p110β may indicate that both catalytic subunits are required to generate sufficient PIP3 to pass some threshold level for downstream signaling. Such a scenario has been proposed previously whereby p110β activity serves to set the threshold for p110α activation (Ciraolo et al., 2008; Jia et al., 2008; Knight et al., 2006). Nevertheless, there must be divergent downstream signaling pathways activated following a rise in PIP3 to explain the opposing PI3K-dependent electrical responses seen in some POMC neurons to leptin and insulin, which results in either K_ATP_ channel activation (hyperpolarization) or the activation of an, as yet, ill-defined putative nonselective cation channel (depolarization). The insulin-mediated hyperpolarization of AgRP neurons observed on genetic deletion of either p110 subunit, or by pharmacological inhibition of PI3K activity, indicates the presence of a separate, possibly PIP3-independent, pathway leading to activation of K_ATP_ channels that is normally occluded by PI3K signaling in these neurons.

Recent studies have begun to link different physiological properties with the various PI3K catalytic subunit isoforms (Shaywitz et al., 2008). For example, we have demonstrated that p110α, and not p110β, plays a key role in developmental angiogenesis (Graupera et al., 2008) and have provided evidence that p110β lies downstream of G protein-coupled receptors (GPCRs) in fibroblasts and macrophages and is not acutely regulated by receptor tyrosine kinases (Guillermet-Guibert et al., 2008). p110α appears to predominantly mediate the effects of insulin and other receptor tyrosine kinases in some tissues, although here we show that p110β is equally important for insulin-mediated changes in electrical excitability of hypothalamic neurons. At present, it is unknown which isoform is responsible for leptin-mediated PI3K actions in tissues, although in this study p110β plays the principal role in POMC neurons. Recently it has been demonstrated that liver-specific deletion of p110β abrogates insulin action in this tissue (Jia et al., 2008). Interestingly, these mice also displayed hyperleptinemia and dysregulation of key hepatic metabolic genes (Jia et al., 2008). Furthermore, these studies suggested that p110β lipid kinase activity is not able to direct PKB phosphorylation but plays a role in S6 kinase-1 activation (Jia et al., 2008), an event that has recently been implicated in hypothalamic regulation of energy homeostasis (Woods et al., 2008). The predominance of p110β signaling, perhaps recruited by GPCRs rather than by IRS2-associated p110α signaling, in POMC and AgRP neurons is consistent with our findings that mice lacking *Irs2* in these cell types have no energy homeostasis phenotype (Choudhury et al., 2005). It is also possible that p110β is playing a scaffolding role in signaling (Ciraolo et al., 2008; Jia et al., 2008) in POMC and AgRP neurons and that this may mediate some of the observed effects of deleting this molecule in these neurons. These considerations may also underlie the ability of PI3K to mediate opposing responses to leptin and insulin in POMC neurons and affect an entirely opposite outcome with respect to excitability observed on the loss or inhibition of one of the isoforms. Furthermore, if these PI3K isoforms are capable of being modulated independently by extrinsic factors, this may profoundly affect the cellular outcome to leptin and insulin and have a major impact on energy homeostasis.

In summary, our studies have revealed an isoform-specific role for class IA PI3K signaling in POMC neurons and demonstrate for the first time the key role of class IA PI3K signaling in AgRP neurons in the long-term regulation of energy homeostasis.

## Experimental Procedures

### Mice and Animal Care

The generation and genotyping of *POMC-Cre* and *AgRP-Cre* (Choudhury et al., 2005; Claret et al., 2007; Xu et al., 2005a, 2005b), *Pik3ca^flox^* or p110α^flox^ (Graupera et al., 2008), and *Pik3cb^flox^* or p110β^flox^ (Guillermet-Guibert et al., 2008) mice have been previously described. Mice with floxed alleles were intercrossed with the indicated *Cre*-expressing transgenic mice to generate compound heterozygote mice. These double heterozygote mice were then intercrossed with *lox^+/−^* mice to obtain WT, *flox^+/+^*, *Cre*, and *Cre/flox^+/+^* mice for each line. To generate mice lacking floxed alleles but expressing GFP or YFP in cells harboring the deletion event, mice were intercrossed with *Z/EG* (Novak et al., 2000) or *Rosa26YFP* (Srinivas et al., 2001) indicator mice and bred to homozygosity for the floxed allele. For detection of Cre-mediated excision of exons 18 and 19 of p110α in the hypothalami of AgRPp110α null and POMCp110α null mice, genomic DNA was isolated from the hypothalamus, cortex, and other tissues of control and mutant mice as previously described (Choudhury et al., 2005). The generation of a 544 bp DNA product following PCR with primers ACACACTGCATCAATGGC and GCTGCCGAATTGCTAGGTAAGC is indicative of excision of the floxed p110α exons in AgRP and POMC neurons of AgRPp110α null and POMCp110α null mice, respectively. For detection of Cre-mediated excision of exons 21 and 22 of the p110β catalytic domain in the hypothalami of AgRPp110β null and POMCp110β null mice, mRNA was extracted from the hypothalamus and cortex of control and mutant mice and transcribed into cDNA as previously described (Choudhury et al., 2005). Resulting cDNA was used as a template for PCR to amplify exons 19–23 using primers located in exon 19 (TTGGACCTGCGGATGCTCCCCTAT) and exon 23 (CGCATCTTCACAGCACTGGCGGA). The generation of a 204 bp PCR fragment in hypothalamic samples from AgRPp110β null and POMCp110β null mice indicated successful splicing of exon 20 onto exon 23, resulting in the generation of an internally truncated p110β protein in these neuronal populations. All knockout and transgenic mice were studied with appropriate littermates of the three control genotypes in all studies. We did not detect any sexual dimorphism in the observed phenotypes. Any mouse that tested positive for deletion in tail tissue due to potential germline recombination of the floxed alleles was excluded from all studies. Mice were maintained on a 12 hr light/dark cycle with free access to water and standard mouse chow (4% fat, RM1, Special Diet Services) and housed in specific pathogen-free barrier facilities. Mice were handled and all in vivo studies performed in accordance to the United Kingdom Animals (Scientific Procedures) Act (1986).

### Metabolic Studies

Body weight and fat measurement, feeding, and HFD studies were performed as previously described (Choudhury et al., 2005; Claret et al., 2007). Plasma leptin and insulin levels were determined using mouse ELISAs (Linco Inc.). MRI scanning was performed as previously described (Choudhury et al., 2005).

### I.c.v. Leptin Treatment

Stainless steel cannulae were inserted into the third ventricle (midline 0 mm, 0.82 mm posterior from bregma, depth 4.8 mm from skull surface) of mice anaesthetized with isoflurane. Postsurgery, mice were singly housed and given at least a week to recover to their presurgery weight. Correct cannula placement was confirmed by demonstration of increased drinking after i.c.v. administration of angiotensin (10 ng). For i.c.v. leptin studies, food was removed from mice 4 hr prior to the onset of the dark phase, and a bolus i.c.v. injection of leptin (0.5 μg) or artificial cerebrospinal fluid (aCSF) was administered. Mice were returned to their home cages immediately after injection. Prior to the onset of dark phase, food was returned and food intake measured at 4 and 24 hr postinjection. All injections were done with an internal cannula projecting 0.5 mm below the tip of the cannula.

### Quantitative RT-PCR Analysis

Quantitative RT-PCR was performed as previously described (Claret et al., 2007). Proprietary sequence Taqman Gene Expression assay FAM/TAMRA primers (Applied Biosystems, Foster City, CA, USA) were used: *Agrp* (Mm00475829_g1), *Hprt* (Mm00446968_m1), *Npy* (Mm00445771_m1), and *Pomc* (Mm00435874_m1).

### Lipid Kinase Assays

PI3K activity assays on hypothalamic lysates were performed as previously described (Bilancio et al., 2006).

### Electrophysiology

Hypothalamic coronal slices (350 μm) were cut from 6- to 18-week-old transgenic mice expressing POMCCreZ/EG or AgRPCreRosa26YFP with or without p110α or p110β mutant alleles. Slices were maintained at room temperature (22°C–25°C) in an external solution containing (in mM) NaCl 125, KCl 2.5, NaH_2_PO_4_ 1.25, NaHCO_3_ 25, CaCl_2_ 2, MgCl_2_ 1, D-glucose 10, and D-mannitol 15 equilibrated with 95% O_2_, 5% CO_2_ (pH 7.4). POMC and AgRP neurons were visualized in the ARC by the expression and excitation of GFP and YFP, respectively. Whole-cell current-clamp (I_fast_) recordings were made at ∼35°C using borosilicate glass pipettes (4–8 MΩ) containing (in mM) Kgluconate 130, KCl 10, EGTA 0.5, NaCl 1, CaCl_2_ 0.28, MgCl_2_ 3, Na_2_ATP 3, GTP 0.3, phosphocreatine 14, and HEPES 10 (pH 7.2), as previously described (Choudhury et al., 2005; Claret et al., 2007; Smith et al., 2007). Following a minimum of 10 min of stable recording, hormones were applied for 2–3 min using a broken tipped pipette (∼3 μm) positioned above the recording neuron. Stock reagents were diluted (≥1000-fold) in a modified external solution with NaHCO_3_ replaced with HEPES (10 mM, pH 7.4). Stocks of recombinant leptin (R&D Systems), insulin (Novo-Nordisk Inc.), TGX-221 (Cayman Chemical Inc.), and wortmannin (Calbiochem Inc.) were diluted in HEPES-buffered external or internal solutions. All other reagents were purchased from Sigma-Aldrich.

### Statistical Analysis

Data are expressed as mean ± SEM. P values were calculated using nonparametric (Mann-Whitney U test) and parametric (unpaired and paired t tests) tests, performed as appropriate. P values ≤0.05 were considered statistically significant. Statistical significance was calculated from all recordings (responsive and nonresponsive) using a Student's two-tailed paired t test or ANOVA, followed by Bonferroni's post hoc test where appropriate.

Additional experimental procedures are presented in the Supplemental Data.

## Figures and Tables

**Table 1:** Effects of Leptin and Insulin on POMC and AgRP Neuron Excitability

	Total		Responsive	
	ΔVm (mV)	Δ Spike (Hz)	ΔVm (mV)	Δ Spike (Hz)
+Leptin				
POMC	+1.3 ± 0.6^∗^ (21)	+0.5 ± 0.3 (21)	+4.5 ± 1.3 (6/21)	+1.6 ± 0.4 (6/21)
POMCp110α null	+3.2 ± 1.4^∗^ (13)	+1.0 ± 0.5^∗^ (13)	+6.3 ± 1.9 (7/13)	+2.2 ± 0.5 (7/13)
POMCp110β null	−2.1 ± 1.1 (17)	+0.1 ± 0.4 (17)	−6.1 ± 1.7 (7/17)	−1.2 ± 0.5 (7/17)
POMC + TGX-221	−0.9 ± 1.6 (11)	+0.3 ± 0.8 (11)	−16.0 (1/11)	−5.6 (1/11)
AgRP	−0.1 ± 1.1 (7)	−0.5 ± 0.8 (7)	N.R.	N.R.
AgRPp110α null	−1.2 ± 0.9 (7)	−0.9 ± 0.4 (7)	N.R.	N.R.
AgRPp110β null	0.0 ± 0.2 (7)	−0.3 ± 0.4 (7)	N.R.	N.R.

+Insulin				

POMC	−4.4 ± 1.1^∗^ (19)	−2.2 ± 0.7^∗^ (19)	−7.1 ± 1.1 (12/19)	−3.4 ± 0.9 (12/19)
POMCp110α null	−0.6 ± 0.3 (8)	−0.2 ± 0.2 (8)	N.R.	N.R.
POMCp110β null	+0.4 ± 0.9 (9)	+1.0 ± 0.6 (9)	N.R.	N.R.
POMC +TGX-221	+0.9 ± 0.6 (8)	−0.1 ± 0.2 (8)	N.R.	N.R.
POMC + PI-103	+1.1 ± 0.7 (7)	+0.3 ± 0.6 (7)	N.R.	N.R.
AgRP	+1.4 ± 0.5^∗^ (13)	+0.5 ± 0.3 (13)	+3.8 ± 0.9 (4/13)	+0.9 ± 0.3 (4/13)
AgRPp110α null	−4.0 ± 0.6^∗^ (7)	−1.9 ± 0.9 (7)	−4.0 ± 0.6 (7/7)	−1.9 ± 0.9 (7/7)
AgRPp110β null	−2.7 ± 1.1^∗^ (11)	−0.4 ± 0.3 (11)	−5.2 ± 1.4 (6/11)	−0.6 ± 0.4 (6/11)
AgRP + wortmannin	−2.0 ± 0.8^∗^ (10)	−0.4 ± 0.3 (10)	−4.2 ± 0.8 (5/10)	−1.1 ± 0.3 (5/10)

Changes in membrane potential (Vm) and spike firing frequency are shown for control, p110α, or p110β null POMC and AgRP neurons. PI3K activity was also blocked in POMC and AgRP neurons by PI-103 (100 nM), wortmannin (100 nM), or the p110β-selective inhibitor TGX-221 (1 μM). Statistical significance (^∗^p < 0.05) was determined from all neurons (Total), irrespective of their response. For qualitative purposes only, responsive neurons were distinguished by a change in Vm greater than ±2 mV. N.R., no apparent response. Numbers of cells are shown in parentheses. Data are expressed as mean ± SEM.

## Supplemental Data

Document S1. Supplemental Experimental Procedures, 12 Figures, 3 Tables, and Supplemental References
