# Supplementary material for: Dominant Role of the p110β Isoform of PI3K over p110α in Energy Homeostasis Regulation by POMC and AgRP Neurons
Source: Cell Metab. 2009 Nov 4;10(5):343–54. doi: 10.1016/j.cmet.2009.09.008 (PMC2806524; doi:10.1016/j.cmet.2009.09.008)
Supplement: Document S1. Supplemental Experimental Procedures, 12 Figures, 3 Tables, and Supplemental References [file mmc1.pdf]

Cell Metabolism, Volume *10*

Supplemental Data

Dominant Role of the p110β Isoform of PI3K over p110α in Energy Homeostasis Regulation by POMC and AgRP Neurons

Hind Al-Qassab, Mark A. Smith, Elaine E. Irvine, Julie Guillermet-Guibert, Marc Claret, Agharul I. Choudhury, Colin Selman, Kaisa Piipari, Melanie Clements, Steven Lingard, Keval Chandarana, Jimmy D. Bell, Gregory S. Barsh, Andrew J.H. Smith, Rachel L. Batterham, Michael L.J. Ashford, Bart Vanhaesebroeck, and Dominic J. Withers

Supplemental Experimental Procedures

Metabolic studies. Blood glucose was measured using a Glucometer Elite (Bayer). Corticosterone levels were measured at early light phase using an Octeia corticosterone EIA (IDS Ltd). Glucose tolerance tests were performed on mice after a 16 h overnight fast. Mice were injected i.p. with D-glucose (1.5 g/kg) and blood glucose levels determined by a glucometer at the indicated time points. For MT-II treatment studies, overnight fasted mice were injected with 50 μg of MT-II (Bachem UK) or vehicle at 08.00 h and food intake monitored for 24 h.

Measurement of metabolic rate. Resting metabolic rate (RMR) of AgRPp110β null and POMCp110β null mice was measured at thermoneutral temperature by open-flow respirometry in male mice using a paramagnetic oxygen analyzer (Series 1100, Servomex) and an infrared carbon dioxide analyzer (Series 1400, Servomex). RMR (ml O_2/min) was converted to its energy equivalents (kJ/day) using the relevant equations (Speakman, 2000; Weir, 1949). The RMR of AgRPp110α null and

POMCp110α null mice was measured by indirect calorimetry using an OXYMAX system (Columbus Instruments Inc.) as previously described (Choudhury et al., 2005).

Hypothalamic immunohistochemistry (IHC) and in situ hybridization (ISH). IHC and ISH were performed as previously described (Choudhury et al., 2005). Rabbit anti-POMC precursor antibody (Phoenix Pharmaceuticals Inc.) was used to detect POMC neurons. ISH ribo-probes against NPY were generated using the mouse sequence (accession number NPY: NM_023456). For co-localisation studies involving p110 and POMC, mice expressing LacZ under the control of the *Pik3ca* or *Pik3cb* promoters were used. Dual staining was performed a rabbit anti-POMC precursor antibody and a mouse monoclonal anti-βGal antibody (Cell Signaling, MA, USA). For quantification of leptin-induced pSTAT-3 generation in POMC and AgRP neurons, 24-h fasted POMCCreZ/eg, **POMCp110**β nullZ/eg, AgRPCreRosa26YFP and AgRP**p110**β nullYFP mice were injected i.p. with either saline or 5μg/g mouse recombinant leptin (R&D Systems). One hour after injection, mice were perfused transcardially and IHC was performed using rabbit an anti-pSTAT3 antibody (Cell Signaling, MA, USA). Imaging was performed with an Olympus BX51 microscope with either a Hamamatsu 95 black and white camera or a Jenoptik PrgRsC14 colour camera combined with SimplePCI capture and deconvolution software.

Neuron counts and assessment of neuronal area. Brains from 16-24 week-old control, P**OMCp110**α null**,** **POMCp110**β null**,** AgRP**p110**α null**, and** AgRP**p110**β null mice were processed as previously described (Choudhury et al., 2005) and cut in 30 μm coronal sections on a microtome. Analysis of neuronal number and area was undertaken as previously described (Claret et al., 2007). In brief, sections throughout the ARC (Bregma –1.1 mm to –2.7 mm) were collected in three series. The distribution and number of POMC or AgRP neurons were counted from one series. To

estimate the total cell number we multiplied neuron count by three to account for the three series. Average somatic area and diameter were analyzed in at least 500 POMC neurons and AgRP neurons. The area occupied by POMC and AgRP neurons was manually scored using SimplePCI software.

Ex vivo hypothalamic slice experiments. Hypothalamic explants studies were performed as previously described (Claret et al., 2007). In brief, mice were killed by cervical dislocation and the whole brain was removed, mounted with the ventral surface uppermost, and placed in a vibrotome A 2.0-mm slice was taken from the base of the brain to include the paraventricular nucleus and the ARC and immediately transferred to artificial cerebrospinal fluid (aCSF) equilibrated with 95% O_2/5% CO_2 and maintained at 37°C. After an initial 2 h equilibration period, the hypothalami were incubated for 45 min in aCSF (basal period). The viability of the tissue was verified by a 45 min exposure to 56 mM KCl. At the end of each period, the aCSF was removed and frozen until being assayed for α-MSH or AgRP by radioimmunoassay (Phoenix Pharmaceuticals Inc.). Hypothalamic explants that failed to show peptide release in response to KCl 3 times greater than that of the basal period were excluded from analysis.

Supplemental References

Choudhury, A.I., Heffron, H., Smith, M.A., Al-Qassab, H., Xu, A.W., Selman, C., Simmgen, M., Clements, M., Claret, M., Maccoll, G., Bedford, D.C., Hisadome, K., Diakonov, I., Moosajee, V., Bell, J.D., Speakman, J.R., Batterham, R.L., Barsh, G.S., Ashford, M.L., and Withers, D.J. (2005). The role of insulin receptor substrate 2 in hypothalamic and beta cell function. The Journal of clinical investigation 115, 940-950.

Claret, M., Smith, M.A., Batterham, R.L., Selman, C., Choudhury, A.I., Fryer, L.G., Clements, M., Al-Qassab, H., Heffron, H., Xu, A.W., Speakman, J.R., Barsh, G.S., Viollet, B., Vaulont, S., Ashford, M.L., Carling, D., and Withers, D.J. (2007). AMPK is essential for energy homeostasis regulation and glucose sensing by POMC and AgRP neurons. The Journal of clinical investigation 117, 2325-2336.

Speakman, J.R. (2000). The cost of living: Field metabolic rates of small mammals. Advances in Ecological Research 30, 177-297.

Weir, J.B.d.V. (1949). New methods for calculating metabolic rate with special reference to protein metabolism. Journal of Physiology 109, 1-9.

Supplemental Figure S1

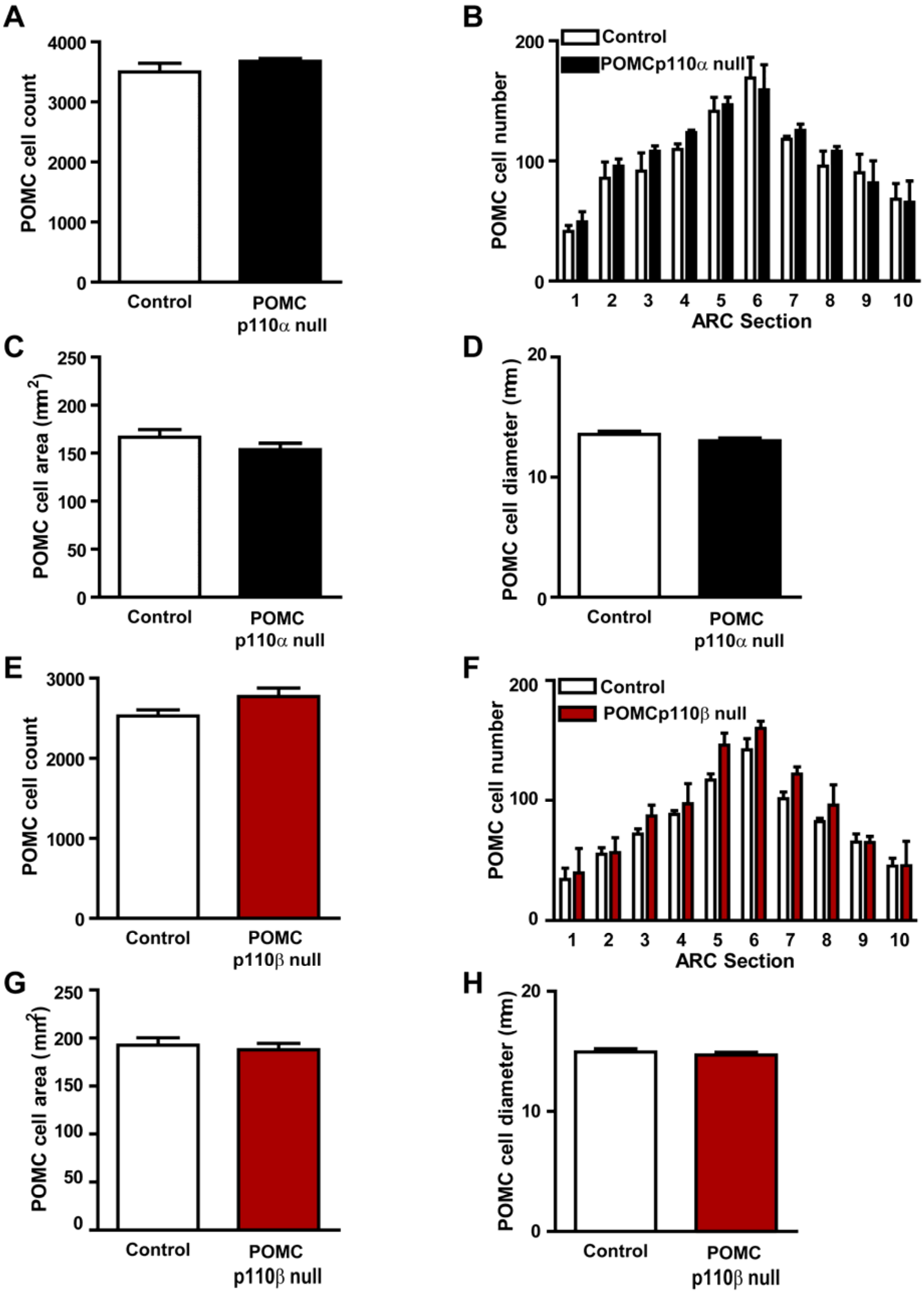

Figure S1. Normal anatomy of POMC neurons in POMCp110α null and POMCp110β null mice. Population size and distribution (**A** and **B**) for POMC neurons within the ARC of control and POMCp110α null mice, n=3. POMC somatic area (**C**) and diameter (**D**) in control and POMCp110α null mice, n=3. Population size and distribution (**E** and **F**) for POMC neurons within the ARC of control and POMCp110β null mice, n=3. POMC somatic area (**G**) and diameter (**H**) in control and POMCp110β null mice, n=3. A minimum of 500 neurons were analyzed per group. All values are mean ± SEM.

Supplemental Figure S2

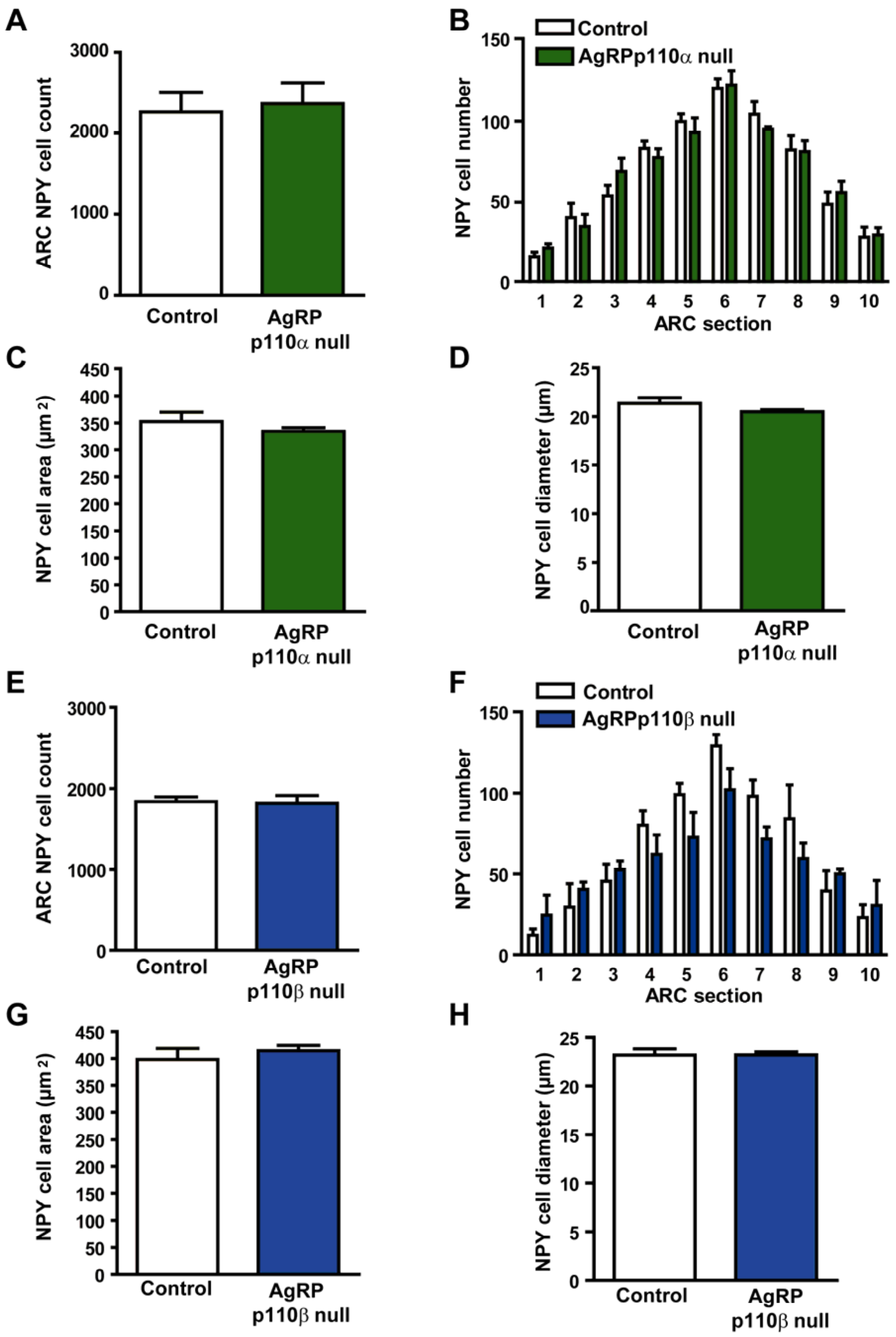

Figure S2. Normal anatomy of AgRP/NPY neurons of AgRPp110α null mice and AgRPp110β null mice. Population size and distribution (**A** and **B**) for AgRP/NPY neurons within the ARC of control and AgRPp110α null mice, n=3. NPY somatic area (**C**) and diameter (**D**) in control and AgRPp110α null mice, n=3. Population size and distribution (**E** and **F**) for AgRP/NPY neurons within the ARC of control and AgRPp110β null mice, n=3. NPY somatic area (**G**) and diameter (**H**) in control and AgRPp110β null mice, n=3. A minimum of 500 neurons were analyzed per group. All values are mean ± SEM.

Supplemental Figure S3

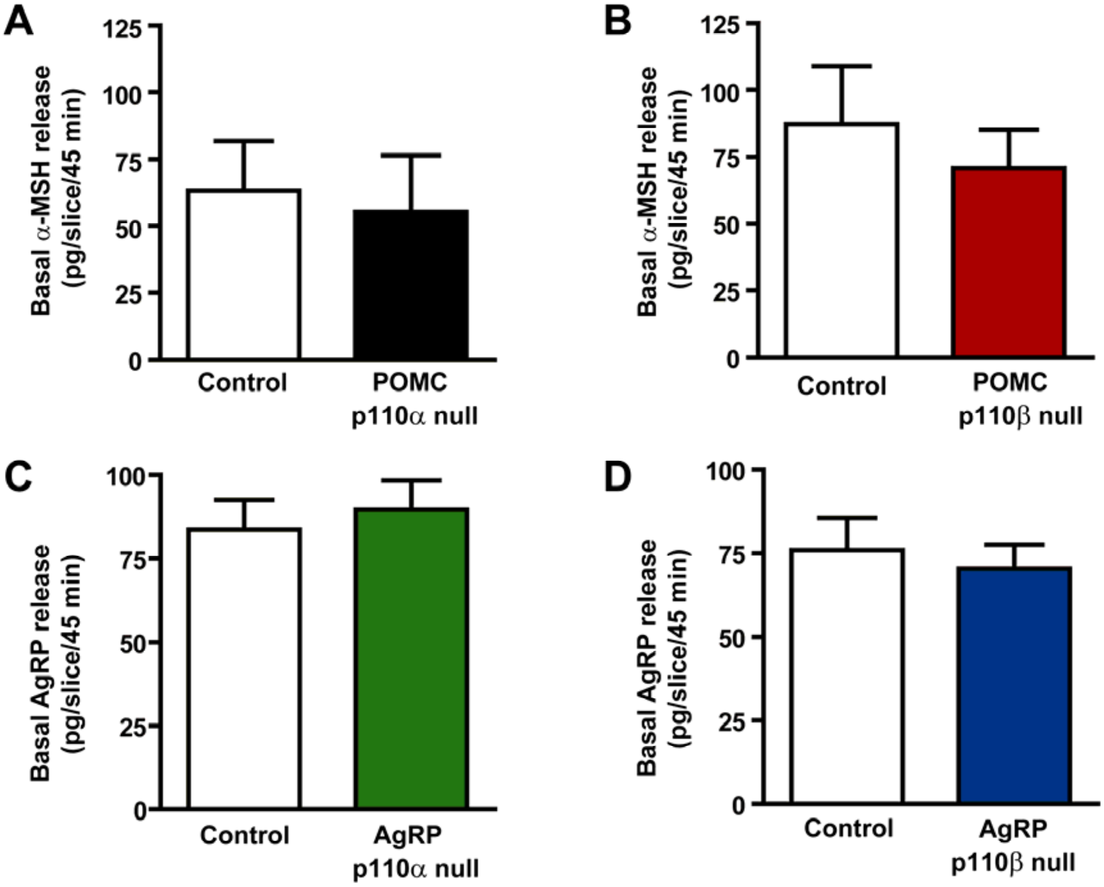

Figure S3. Unaltered neuropeptide release in hypothalamic explants from POMCp110 null and AgRPp110 null mice. α-MSH release from hypothalamic explants of POMCp110α null (**A**) and POMCp110β null (**B**) mice**,** n=6. AgRP release from hypothalamic explants of AgRPp110α null (**C**) and AgRPp110β null (**D**) mice, n=6. All values are mean ± SEM.

Supplemental Figure S4

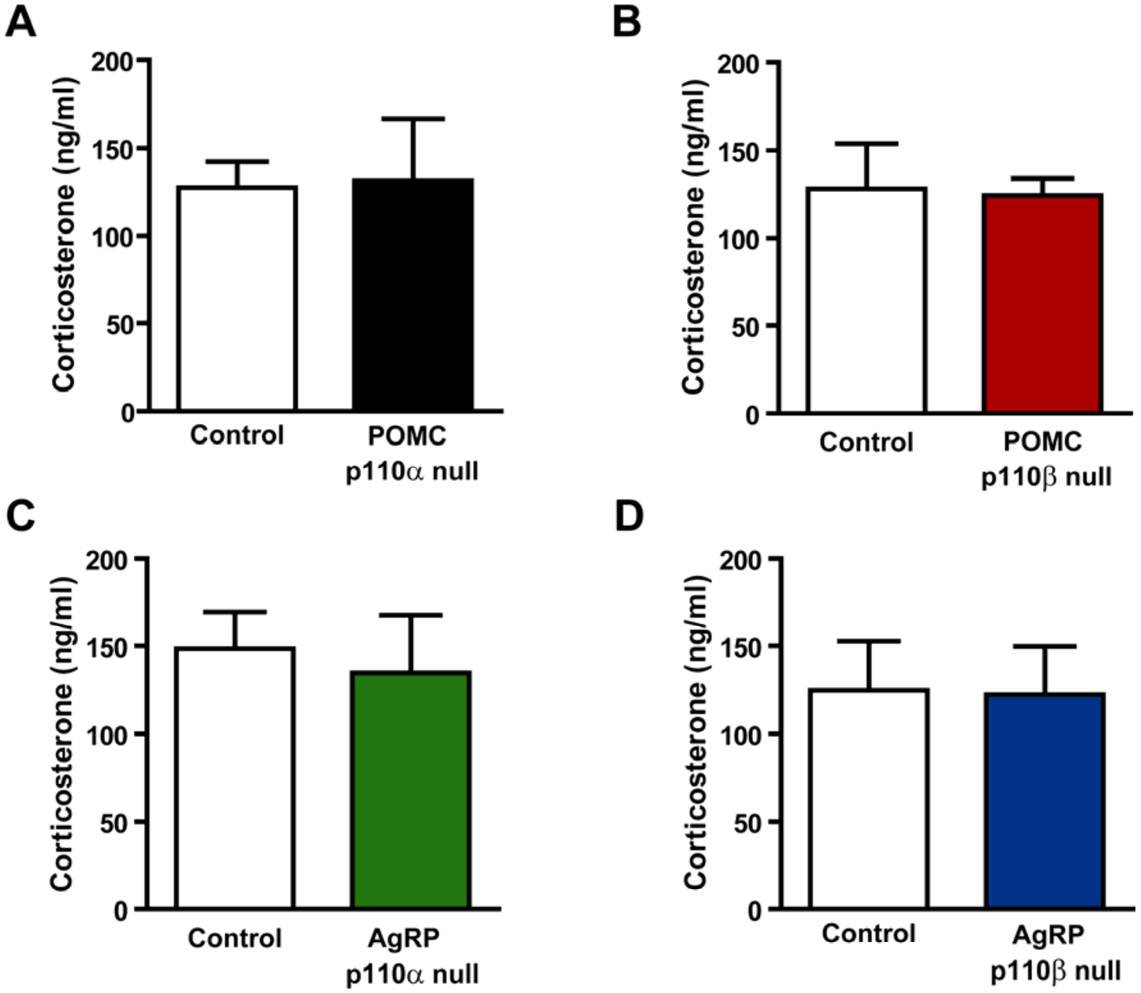

Figure S4. Normal basal corticosterone levels in POMCp110 null and AgRPp110 null mice. Plasma corticosterone levels in POMCp110α null (**A**), POMCp110β null (**B**), AgRPp110α null (**C**) and AgRPp110β null (**D**) mice, n=6. All values are mean ± SEM.

Supplemental Figure S5

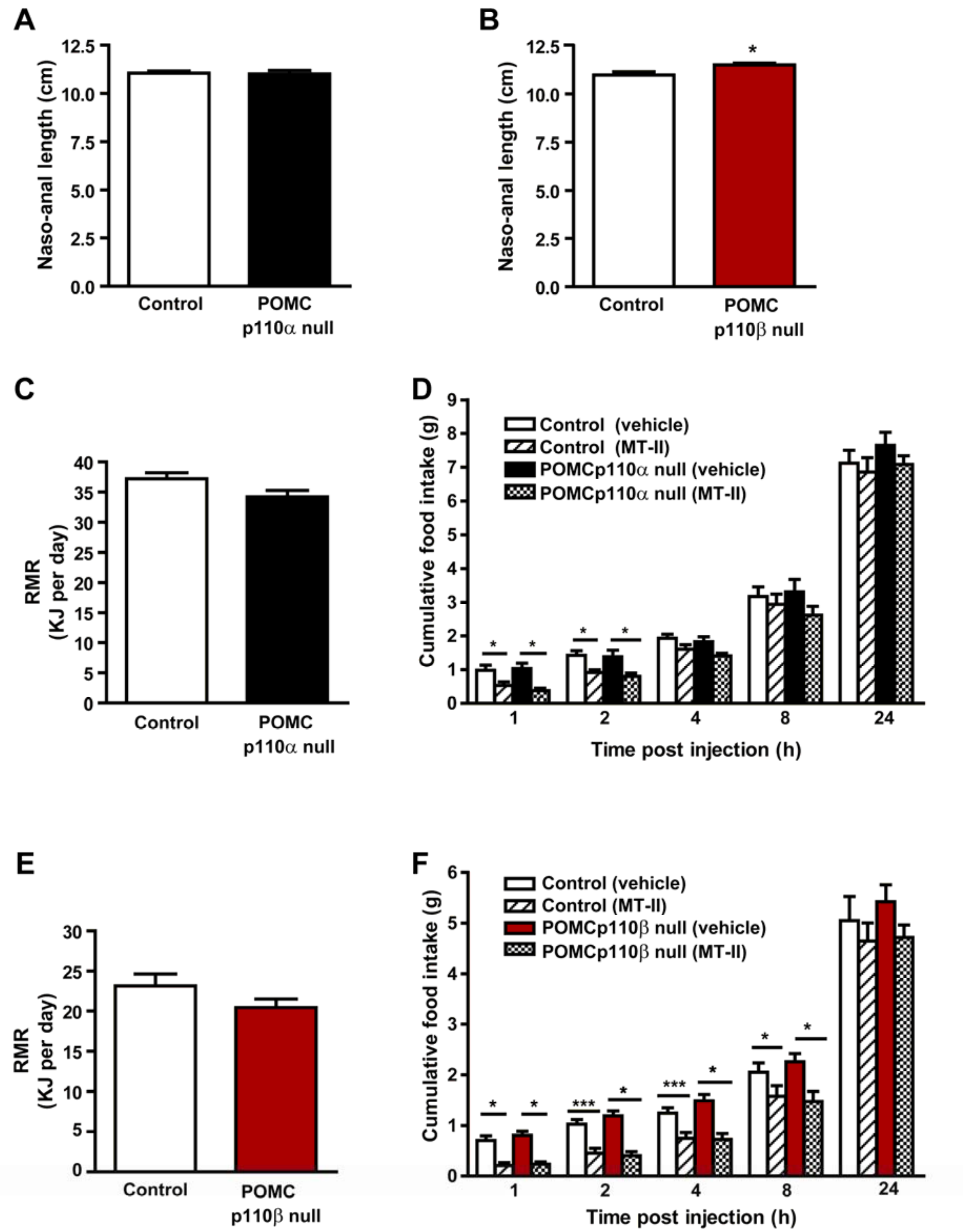

Figure S5. Length, resting metabolic rate (RMR) and sensitivity to MT-II in mice lacking functional p110α and p110β in POMC neurons. Naso-anal body length of 16-week old

POMCp110α null (**A**) and POMCp110β null (**B**) mice, n=7. (**C**) Resting metabolic rate determined by open-flow respirometry in male 16-week-old control and POMCp110α null mice. (**D**) Cumulative food intake at the times indicated after injection of vehicle or MT-II following an overnight fast in 16-week-old male control and POMCp110α null mice. (**E**) Resting metabolic rate determined by open-flow respirometry in 16-week-old control and POMCp110β null mice, n=10. (**F**) Cumulative food intake at the times indicated after injection of vehicle or MT-II following an overnight fast in 16-week-old male control and POMCp110β null mice, n=8. All values are mean ± SEM. * $P<0.05$, *** $P<0.001$.

Supplemental Figure S6

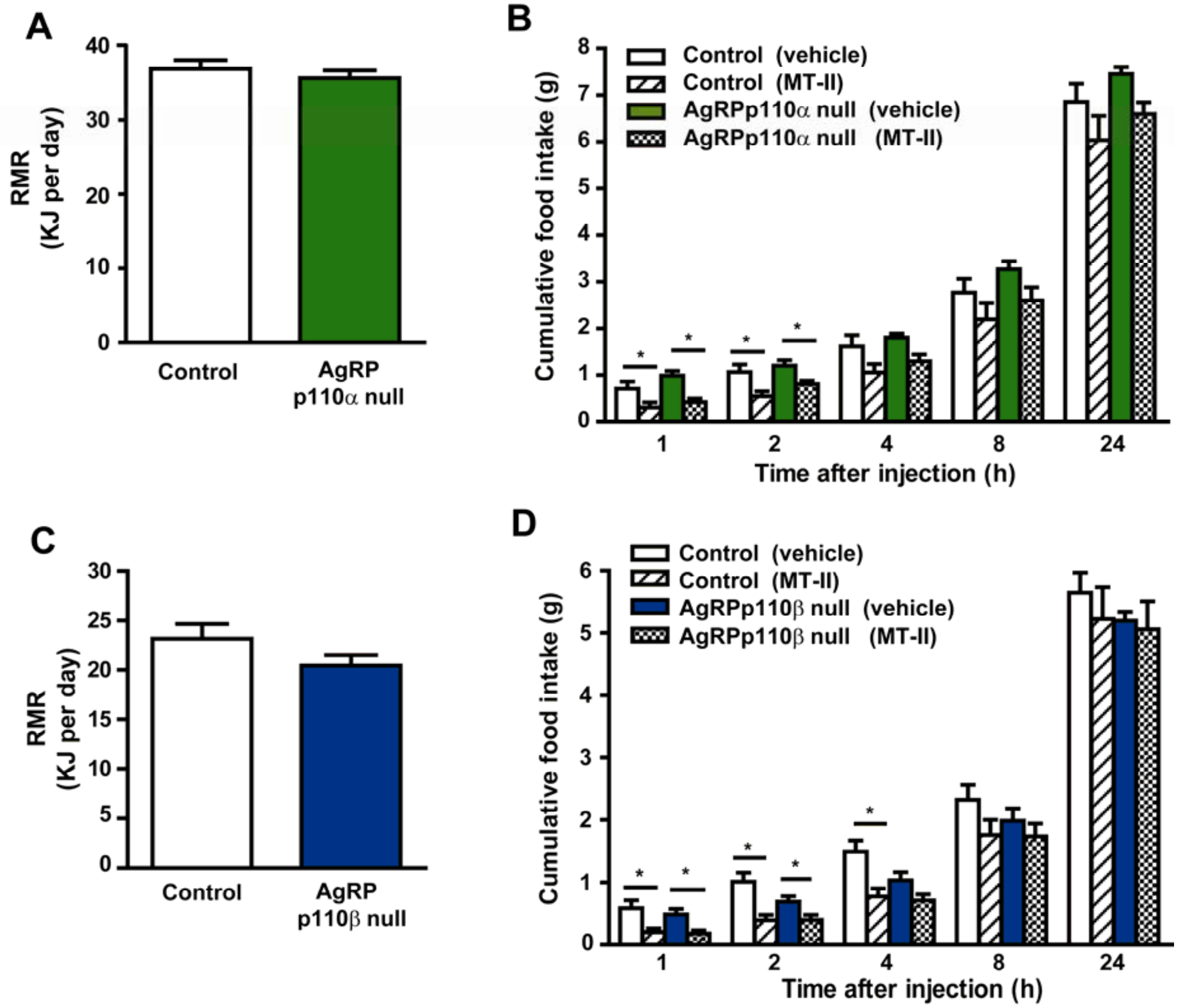

Figure S6. Resting metabolic rate (RMR) and sensitivity to MT-II in mice lacking functional p110α and p110β in AgRP neurons. (**A**) RMR determined by open-flow respirometry in 16-week-old control and AgRPp110α null mice, n=10. (**B**) Cumulative food intake at the times indicated after injection of vehicle or MT-II following an overnight fast in 16-week-old male control and AgRPp110α null mice**,** n=8. (**C**) RMR determined by open-flow respirometry in 16-week-old control and AgRPp110β null mice, n=10. (**D**) Cumulative food intake at the times indicated after injection of vehicle or MT-II following an overnight fast in 16-week-old male control and AgRPp110β null mice**,** n=8. All values are mean ± SEM. * $P < 0.05$.

Supplemental Figure S7

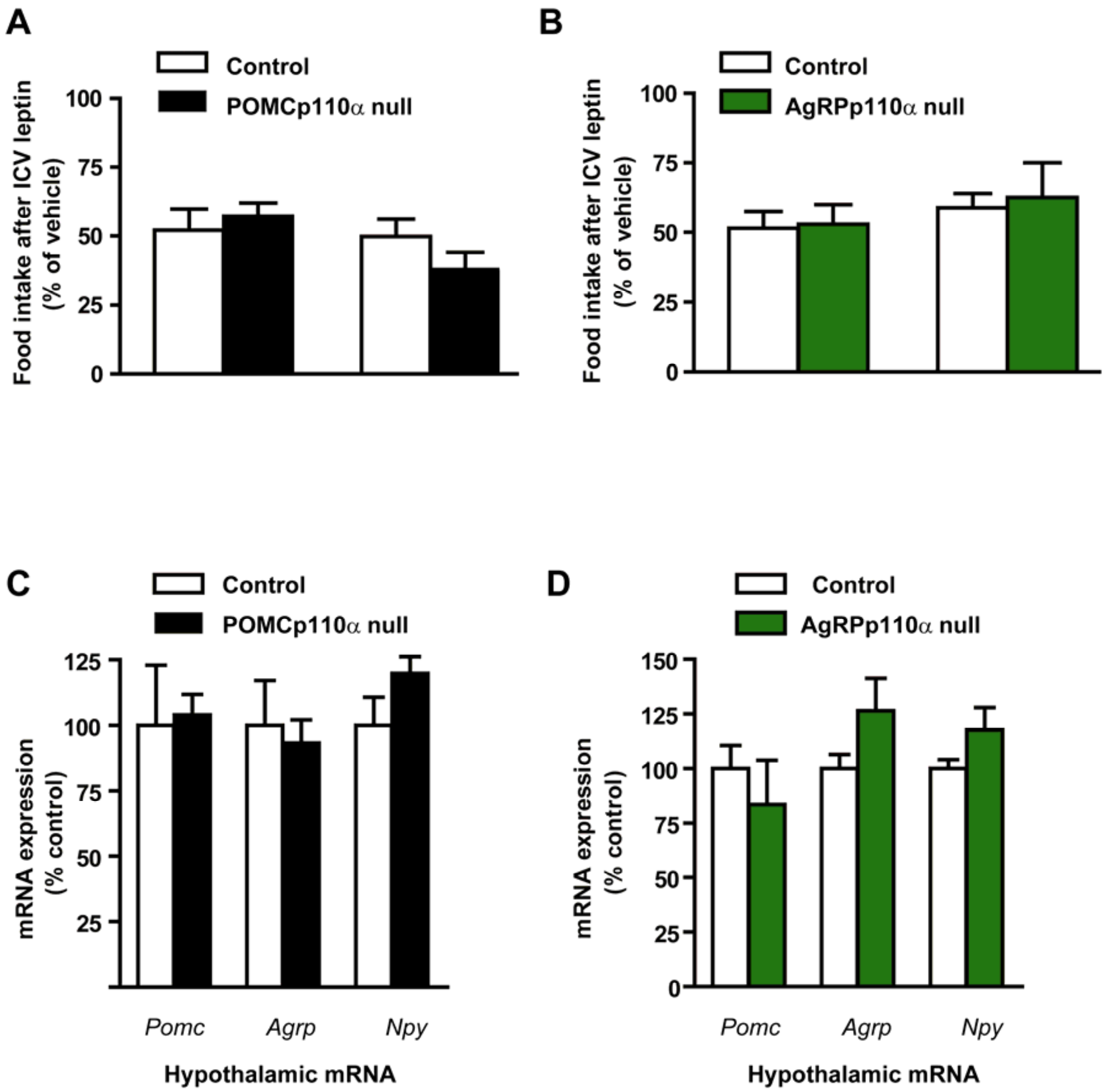

Figure S7. Response to i.c.v. leptin and hypothalamic feeding peptide expression in POMCp110α null and AgRPp110α null mice. Food intake at 4 h and 24 h post i.c.v injection of leptin (0.5 μg) in POMCp110α null (**A**) and AgRPp110α null (**B**) mice, n=8. *Pomc*, *Agrp* and *Npy* mRNA expression in hypothalami of POMCp110α null (**C**) and AgRPp110α null (**D**) mice, as assessed by quantitative RT-PCR, n=8. Probes for HPRT were used to adjust for total RNA content. All values are mean ± SEM.

Supplemental Figure S8

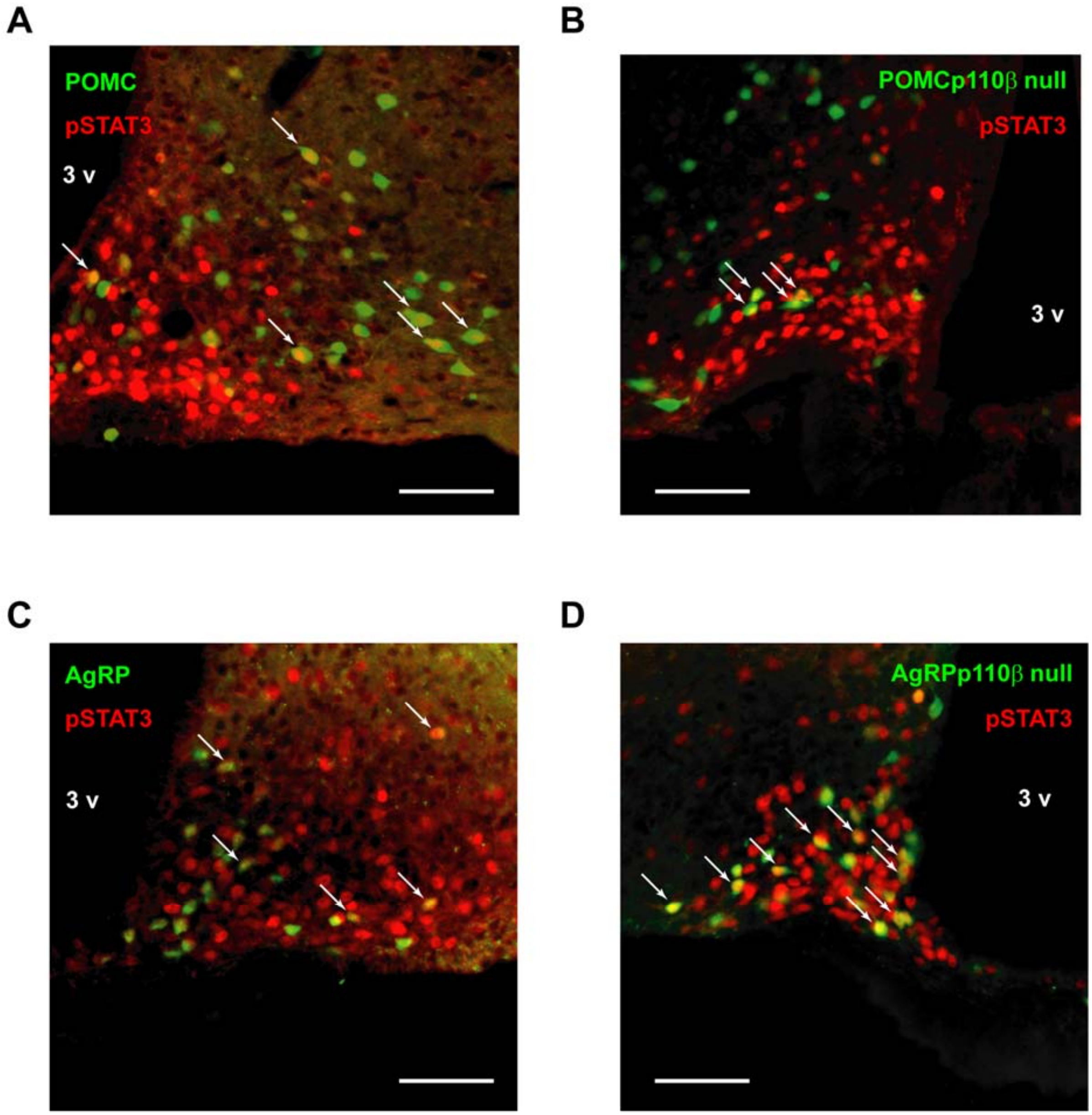

Figure S8. Leptin-induced pSTAT3 generation in POMC and AgRP p110β null neurons. Following a 24-h fast, mice were injected i.p. with 5μg/g of leptin and transcaridally perfused one hour later. IHC quantifcation of the percentage of co-localization of POMC neurons (green) with pSTAT3 (red) demonstrated no differences in leptin-

induced pSTAT3 generation between (A) POMCCreZ/EG (**A**) mice and **POMCp110**β nullZ/EG (**B**) mice. Quantification of the percentage of co-localization of AgRP neurons (green) with pSTAT3 (red) demonstrated no differences in leptin-induced pSTAT3 generation between AgRPCreRosa26YFP (**C**) and AgRP**p110**β nullYFP **(**D**)** mice. Scale bars are 100 μm and arrows represent colocalization.

Supplemental Figure S9

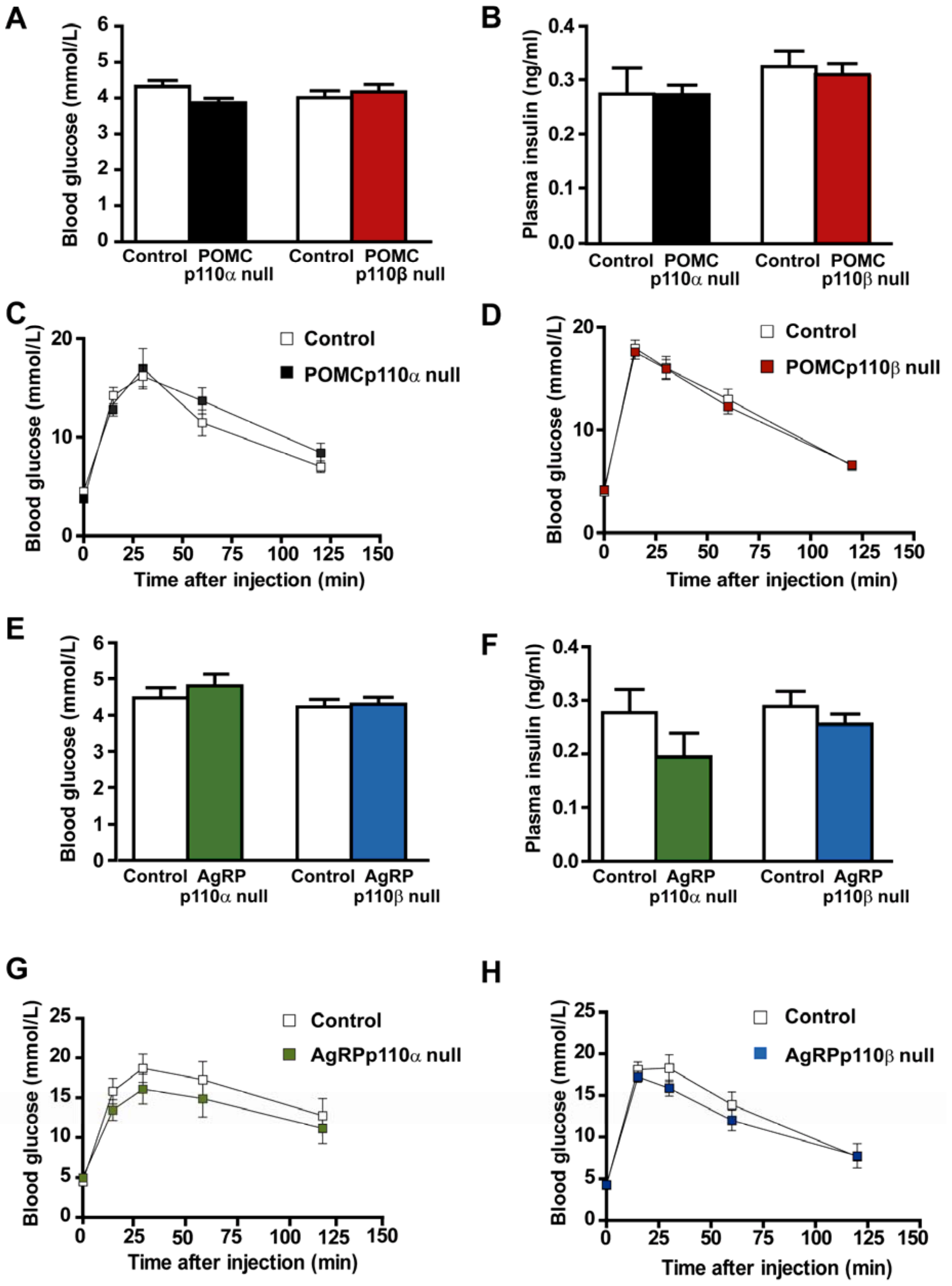

Figure S9. Glucose homeostasis in POMCp110 null and AgRPp110 null mice. (**A**) Fasting blood glucose levels in 16-week old male POMCp110α null and POMCp110β null mice, n=12. (**B**) Fasting insulin levels in 16-week old male POMCp110α null and POMCp110β null mice, n=10. Glucose tolerance in 16-week old male POMCp110α null (**C**) and POMCp110β null (**D**) mice, n=12. (**E**) Fasting blood glucose levels in 16-week old male AgRPp110α null and AgRPp110β null mice, n=12. (**F**) Fasting insulin levels in 16-week old male AgRPp110α null and AgRPp110β null mice, n=10. Glucose tolerance in 16-week old male AgRPp110α null (**G**) and AgRPp110β null (**H**) mice, n=12. All values are mean ± SEM.

Supplemental Figure S10

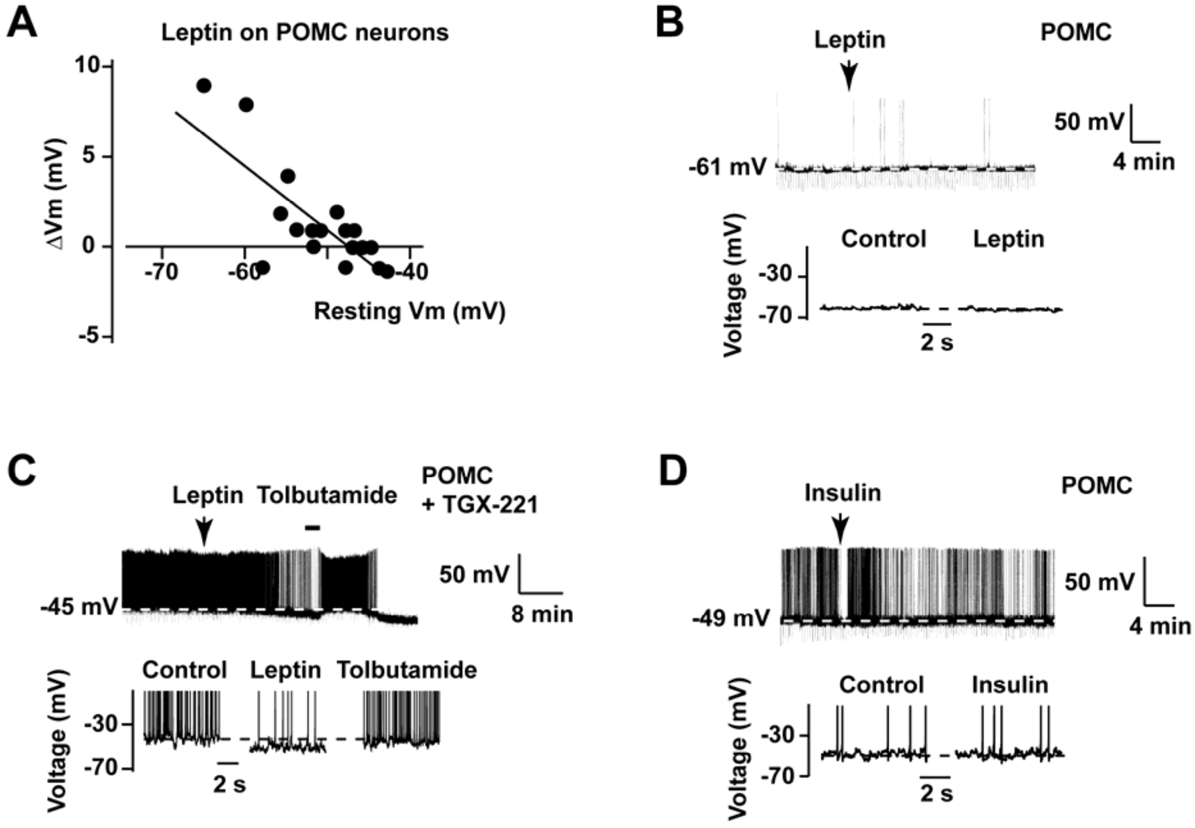

Figure S10. Leptin and insulin modulates a minority of POMC neuronal excitability. **A**, plot of leptin mediated depolarization of POMC neurons against resting membrane potential. The majority of POMC neurons are unresponsive to leptin (**B**) and insulin (**D**). **C**, leptin hyperpolarized a p110β-inhibited (1 μM TGX-221) POMC neuron, an effect that was reversed by bath-applied 200 μM tolbutamide.

Supplemental Figure S11

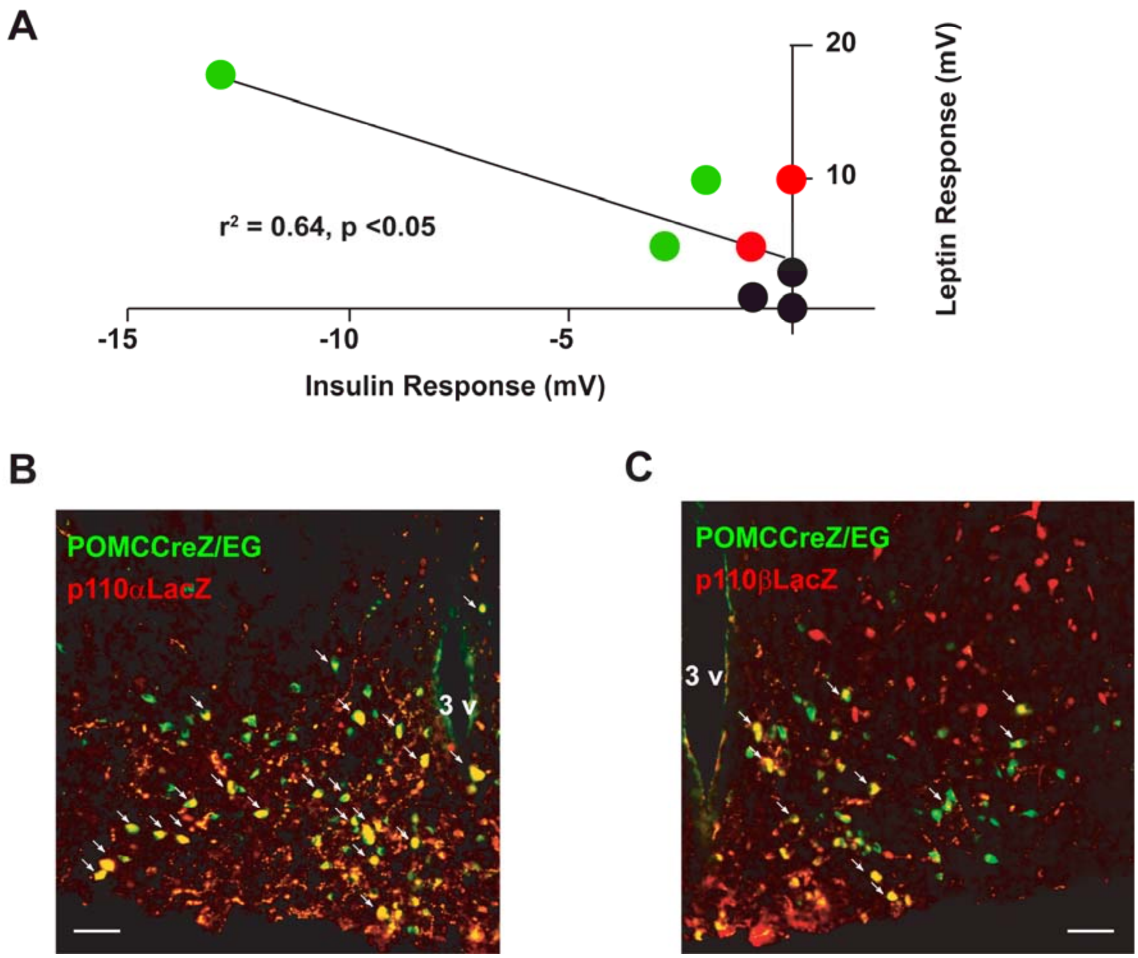

Figure S11. Insulin and leptin modulate individual POMC neurons and expression of p110α and p110β in POMC neurons . **A**, plot of the change in membrane potential (Vm) following sequential insulin and leptin application, where black circles represent no insulin or leptin response, green circles are from neurons that responded to both insulin and leptin, and red circles are from neurons that responded to leptin but not insulin. Data is fitted with linear regression. Imunohistochemistry for LacZ (red) driven by the p110α (**B**) or p110β (**C**) promoters is shown in arcuate neurons expressing GFP driven by the POMC promoter. Scale bars are 100 μm and arrows represent colocalization.

Supplemental Figure S12

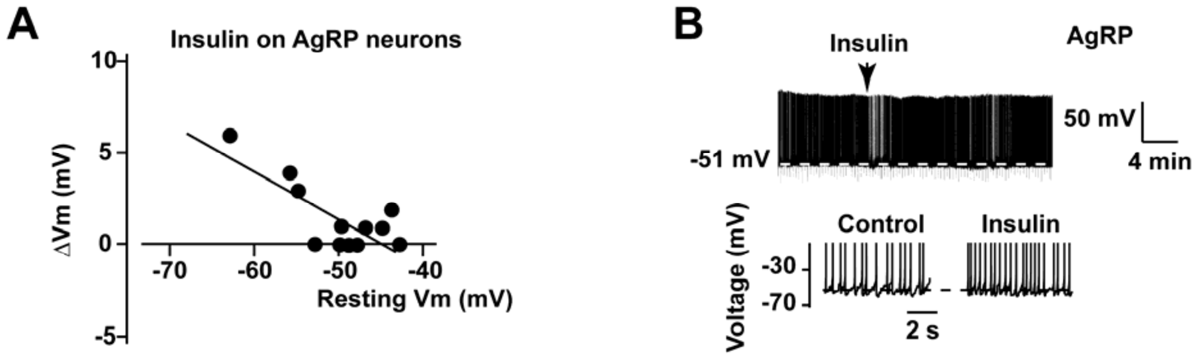

Figure S12. Insulin does not modulate the excitability of all AgRP neurons. **A**, plot of insulin mediated depolarization of AgRP neurons against resting membrane potential. The minority of AgRP neurons are unresponsive to insulin (**B**).

Table S1. Biophysical properties of control, p110α null and p110β null POMC and AgRP neuronal population. Numbers of cells tested are shown in parentheses. Data are expressed as a mean ± SEM. Statistical significance (* *P*<0.05.) between control and p110 mutants was determined by ANOVA with Bonferroni post hoc analysis.

	Membrane Potential (mV)	Input Resistance (GΩ)	Spike Firing Frequency (Hz)	Capacitance (pF)
POMC	-50 ± 1<br>(54)	2.8 ± 0.1<br>(54)	4.3 ± 04<br>(54)	7.2 ± 0.8<br>(10)
POMCp110α null	-56 ± 1*<br>(21)	3.1 ± 0.2<br>(21)	2.0 ± 0.5*<br>(21)	6.5 ± 1.8<br>(5)
POMCp110β null	-50 ± 1<br>(43)	2.9 ± 0.1<br>(43)	3.3 ± 0.4<br>(43)	7.4 ± 0.8<br>(6)
AgRP	-50 ± 1<br>(19)	3.1 ± 0.3<br>(19)	3.8 ± 1.0<br>(19)	7.3 ± 0.9<br>(6)
AgRPp110α null	-48 ± 1<br>(14)	3.2 ± 0.4<br>(14)	4.8 ± 0.6<br>(16)	7.4 ± 0.5<br>(5)
AgRPp110β null	-50 ± 1<br>(18)	3.7 ± 0.4<br>(18)	3.5 ± 0.6<br>(18)	9.5 ± 1.2<br>(5)

Table S2. Metabolic phenotype of mice and excitable properties of control POMC and POMCp110β null neurons. Male littermate mice were singly housed prior to in-vitro electrophysiological recordings. Resting membrane potential (Vm), spike firing frequency and input resistance were not different between the mutants, age, or metabolic phenotype. Change in membrane potential (ΔVm) following leptin (50 nM) application was observed in control but not p110β null POMC neurons. Data is expressed as mean ± SEM. * P < 0.05 from age matched controls. N = 6 mice per group.

	Control (young)	POMCp110β null (young)	Control (older)	POMCp110β null (older)
Age (week)	7.5 ± 0.4	7.7 ± 0.6	18.4 ± 0.4	18.0 ± 0.3
Daily Food Intake (g)	3.0 ± 0.1	3.4 ± 0.1*	3.0 ± 0.2	3.7 ± 0.2*
Vm (mV)	-52 ± 2	-50 ± 1	-50 ± 2	-51 ± 2
Spike Frequency (Hz)	3.3 ± 0.7	4.9 ± 1.3	3.9 ± 0.6	3.6 ± 0.8
Input Resistance (GΩ)	3.0 ± 0.3	2.5 ± 0.3	2.5 ± 0.2	2.6 ± 0.2
Leptin ΔVm (mV)	+2.9 ± 1.3	-2.9 ± 0.9 *	+1.7 ± 0.8	-0.8 ± 0.6*

Table S3. Summary of effects of genetic inactivation of either p110α or p110β in POMC and AgRP neurons. =, unaffected; ↑, increased; ↓, decreased; as compared to control mice. N.A., no affect; hyperpol., hyperpolarization; depol., depolarization; HFD, high fat diet; i.c.v., intracerebroventricular.

		POMCp110α null	POMCp110β null	AgRPp110α null	AgRPp110β null
**Food intake**	*Ad-libitum*	=	↑	=	↓
	**Re-feeding**	=	↑	=	↓
	**Response to i.c.v. leptin**	=	↓	=	↑↑
**Fasting plasma leptin levels**		=	↑	=	↓↓
**Sensitivity to HFD**		↑	↑	=	↓↓
**Resting metabolic rate**		=	=	=	=
**Neuropeptide expression**	*Npy*	=	=	=	↓
	Pomc	=	↓	=	=
**Electrophysiological responses**	**Leptin**	Normal depol.	Hyperpol.	N.A.	N.A.
	**Insulin**	Absent	Absent	Hyperpol.	Hyperpol.
**Overall energy homeostasis phenotype**		Increased sensitivity to HFD	Increased adiposity and hyperphagia<br>Increased sensitivity to HFD	Normal	Lean hypophagic<br>Resistant to HFD
